# Farnesyltransferase inhibitors decrease matrix-vesicle-mediated mineralization in SaOS-2 cells

**DOI:** 10.1007/s11033-025-11138-2

**Published:** 2025-11-19

**Authors:** Tim Bürgel, Daniel Diehl, Elisabeth-Cosima van Lier, Anton Friedmann, Anna Hagemann, Hagen S. Bachmann

**Affiliations:** 1 Institute of Pharmacology and Toxicology, Centre for Biomedical Education and Research, Witten/Herdecke University, Witten, Germany; 2 Department of Periodontology, Centre for Biomedical Education and Research, Witten/Herdecke University, Witten, Germany

**Keywords:** Farnesyltransferase, Mineralization, Osteosarcoma, Tipifarnib, Lonafarnib, Prenylation

## Abstract

**Background:**

Farnesyltransferase inhibitors (FTIs) were developed for preventing the prenylation of aberrant Ras in human cancer. Furthermore, prenylation is involved in multiple biological processes and plays a putative role in signalling pathways such as the Ras-mitogen-activated-protein kinase (MAPK) pathway. Matrix-vesicle-mediated mineralization (MVM) is the first step in the development of eukaryotic mineralized tissue. In this study, we evaluated the effect of FTIs on MVM in the osteosarcoma cell line SaOS-2 and elucidated the role of farnesylation in this process.

**Methods:**

SaOS-2 cells were treated with the FTIs Lonafarnib and Tipifarnib. Mineralization was assessed using Alizarin Red S staining. Enzyme assays were conducted to measure alkaline phosphatase (ALP) activity. Western blot analysis and Oxford Nanopore sequencing were performed to evaluate the expression of ALP, collagen type I (COL1A1), and Runt-related transcription factor 2 (RUNX2).

**Results:**

Inhibition of farnesylation by FTIs resulted in decreased mineralization, as evidenced by reduced Alizarin Red S staining. Additionally, RUNX2 activity was diminished, leading to a reduction in MVM and decreased expression of ALP and COL1A1.

**Conclusion:**

Our findings demonstrate that FTIs Lonafarnib and Tipifarnib impair MVM, highlighting the essential role of farnesylation in biomineralization.

**Supplementary Information:**

The online version contains supplementary material available at 10.1007/s11033-025-11138-2.

## Introduction

Many eukaryotic proteins require post-translational modification (PTM) with a lipid group to facilitate membrane interaction and normal function [1]. Protein prenylation is a major type of PTM and is crucial for various developmental processes, such as early embryogenesis and the progression of cancer [2–4]. Prenylation refers to the addition of an isoprenoid moiety to the C-terminus of a protein via a covalent thioether bond [5]. As a result, increased molecular hydrophobicity facilitates the association with lipid biomembranes or enables adequate protein‒protein interactions [6–8]. The process is catalysed by one of four eukaryotic prenyltransferases, either farnesyltransferase (FTase) (EC 2.5.1.58) or one of three geranylgeranyltransferases [GGTase I-III: GGTase I (EC 2.5.1.59), GGTase II (EC 2.5.1.60) or GGTase III (no EC yet)] [9–11]. All four are heterodimeric enzymes. FTase and GGTase I share a 48 kDa α subunit but differ in their unique β subunits [12]. GGTase II, also known as RabGGTase, and GGTase III, in contrast, share the same β subunit, forming dimers with a unique α subunit [11, 13]. Prenylation is dependent on the mevalonate pathway since these enzymes generate isoprenoid moieties from substrates obtained by farnesyl diphosphate synthase (FDPS). FTase catalyses the attachment of a C15 farnesyl group from its substrate farnesyl pyrophosphate (FPP) to the C-terminus of proteins carrying a so-called CAAX box motif [14]. The most prominent protein is the small GTPase Ras and its isoforms, which are known to require farnesylation to bind to the membrane and therefore fulfil their functions [15]. Activated Ras concentrates effector proteins in signalling cascades, enabling interactions with proteins and lipids to control the downstream mitogen-activated protein kinase (MAPK) pathway, which is known to regulate apoptosis, cell proliferation, migration and cell growth [16, 17].

Inhibitors of FTase (FTIs) were developed as anticancer drugs to pharmacologically inhibit the farnesylation step of those Ras proteins. Many FTIs have been designed, such as Tipifarnib and Lonafarnib, which are competitive inhibitors of the CAAX peptide [18–20]. Both mentioned FTIs are the most studied and promising candidates in clinical trials [21].

The osteogenic differentiation of immature osteoblasts is required to maintain physiological bone homeostasis. Osteoblastic cells are specialized cell types that produce a mineralized extracellular matrix (ECM), which is crucial for the formation of mineralized tissues, a unique feature of the vertebrate skeletodental system [22]. Mineralization of hard tissues in vertebrates involves two phases: primary and secondary mineralization. Primary mineralization, also known as matrix-vesicle-mediated mineralization (MVM), involves extracellular nanoscale vesicles of approximately 50–200 nm [23]. Matrix vesicles (MVs) are budded off by osteoblasts, hypertrophic chondrocytes and odontoblasts, together with large amounts of collagen fibrils and noncollagenous proteins collectively referred to as osteoids [24]. These vesicles are loaded with enzymes and transporters such as ectonucleotide pyrophosphatase/phosphodiesterase and tissue nonspecific alkaline phosphatase (ALP), which promote the hydrolysis of inorganic phosphate compounds to supply monomeric phosphate [25, 26]. Because of primary mineralization, Ca^2+^ and PO_4_^3−^ accumulate inside the vesicle and induce crystal nucleation [27]. Calcium phosphate crystals grow, penetrate the vesicle membrane, and form calcified nodules inside the collagenous ECM [28]. While physiological mineralization predominantly occurs within the body’s hard tissues, such as bones and teeth, ectopic mineralization of soft tissues may result in serious health issues. Ectopic mineralization may occur in chronic diseases such as nephrolithiasis, aortic valve stenosis and osteoarthritis or as a symptom of rare genetic mutations, as observed in pseudoxanthoma elasticum (PXE) or Keutel syndrome [29]. Intriguingly, the molecular dynamics of ectopic mineralization resemble those of its physiological counterpart, as MVM and cells with osteoblastic phenotypes have been frequently observed in calcifying soft tissues [30–32]. This finding is because soft tissue mineralization generally needs to be actively suppressed by inhibitors such as pyrophosphate, fetuin-A or matrix Gla protein, and ectopic mineralization is most commonly associated with pathophysiological loss of these inhibitors [33]. Consequently, therapeutic strategies are aimed at supplementing mineralization inhibitors, phosphate binders or inhibitors of ALP, along with causative therapies for the underlying inflammatory or atherogenic diseases [34]. Interestingly, drugs targeting the mevalonate pathway have been reported to have positive effects on genetic and chronic ectopic mineralization disorders. In this context, statins are not only used routinely as cholesterol-lowering drugs but also have been reported to ameliorate soft tissue mineralization in a PXE mouse model [35]. Moreover, bisphosphonates, which are pyrophosphate analogues and inhibitors of FDPS, ameliorated mineralization more effectively in a similar mouse model and in clinical trials using etidronate [36, 37]. In cardiovascular disease, a recent systematic review reported that bisphosphonates were generally able to reduce vascular mineralization [38]. However, while these treatments were seemingly effective, their long half-life and severe side effects, such as medication-related jaw necrosis, make their routine clinical usage a matter of debate in the scientific community [39]. The reported mechanism of statin- or bisphosphonate-mediated antimineralization effects is centred on their primary pharmacodynamic properties: diminishing FPP levels in the mevalonate pathway, thereby preventing farnesylation of proteins responsible for mineralization-related signalling pathways [40]. This phenomenon is further underlined by reports of reduced osteoblastogenesis following treatment with the farnesyltransferase inhibitor FTI-277 [41]. Unfortunately, FTI-277 has never advanced to clinical studies. Therefore, this study aimed to examine the impact of two clinically used FTIs, Lonafarnib and Tipifarnib, on MVM and thereby further elucidate the role of farnesylation in biomineralization.

## Materials and methods

### Cell culture and treatment

The human osteosarcoma cell line SaOS-2 (ATCC HTB-85) was cultured in Dulbecco’s modified Eagle’s medium (DMEM, PAN Biotech, Aidenbach, Germany) supplemented with 15% (v/v) foetal bovine serum (FBS, PAN Biotech), 100 U/ml penicillin and 100 µg/ml streptomycin (both from PAN Biotech). Upon confluence, the cells were washed in phosphate-buffered saline (PBS, PAN Biotech), detached and seeded into 6-well culture plates at a density of 3 × 10^5^ vital cells per well for consecutive experiments. Before seeding, the adequate cell count and viability were assessed via a fluorescence-based flow cytometric assay (Muse Count & Viability, Merck Millipore, Burlington, Massachusetts, USA). For the induction of mineralization, the medium was further supplemented with 50 µg/ml ascorbic acid (AA; Sigma-Aldrich, Taufkirchen, Germany) and 10 mM β-glycerophosphate (β-GP, Sigma-Aldrich). For FTase inhibition, the osteogenic medium was supplemented with either Tipifarnib or Lonafarnib [10 and 20 µM, dissolved in dimethyl sulfoxide (DMSO, PAN Biotech)]. Drug concentrations were determined experimentally via the MTT assay. The control cultures were treated with an equivalent amount of DMSO. Unless otherwise stated, the cells were incubated in a humidified 5% CO_2_ environment at 37 °C for 72 h. For determination of the effects of the prenyltransferase substrates FPP (Sigma-Aldrich) and geranylgeranylpyrophosphate (GGPP; Sigma-Aldrich) on the mineralization of SaOS-2 cells, confluent monolayers in 6-well plates were treated with different concentrations of exogenous FPP and GGPP dissolved in DMSO. The FPP and GGPP concentrations used were 2 µM, 5 µM, and 10 µM.

### Alizarin red S (ARS) staining assay

Confluent SaOS-2 cell monolayers were washed twice in PBS and fixed in 4% (v/v) formaldehyde (Sigma-Aldrich) at room temperature for 30 min. After the cells were washed twice in dH_2_O, 1 ml of 40 mM ARS (Carl Roth, Karlsruhe, Germany; dissolved in dH_2_O, pH 4.1) was added. The monolayers were stained for 30 min under constant shaking. Afterwards, the unincorporated dye was aspirated, the cells were washed with dH_2_O, and the stained monolayers were visualised with a microscope (Nikon Eclipse TS100) and stored at −20 °C. For dye extraction, 800 µl of 10% (v/v) acetic acid (Carl Roth) was added to each well, and the plates were incubated under constant shaking for 60 min. The monolayers were then collected via a cell scraper (Thermo Fisher Scientific, Massachusetts, USA), transferred to 2 ml microcentrifuge tubes, and vortexed for 60 s. The tubes were heated to 85 °C for 10 min and then cooled on ice for 5 min. The slurry was centrifuged (20,000 × g, 15 min), and 500 µl of each supernatant was adjusted to pH 4.1–4.5 by adding 10% (v/v) ammonium hydroxide (Carl Roth) to a new microcentrifuge tube. Aliquots of 100 µl were read in triplicate at 405 nm in 96-well transparent-bottom plates via a multiplate Tecan reader (Tecan Infinite200 PRO plate reader, Tecan Trading AG, Switzerland).

### Tetracycline staining assay

Confluent monolayers of SaOS-2 cells were washed once in Dulbecco’s balanced salt solution (DPBS, Pan Biotech). A stock of 10 µg/µl tetracycline hydrochloride (Sigma-Aldrich, dissolved in PBS) was added to each well at a volume of 1 ml. The cells were incubated for 48 h at 37 °C and washed twice in DPBS, and the fluorescence signal was measured (16 reading points per well, 390 nm_exc_/560 nm_em_) via a multiple-plate Tecan reader.

### Flow cytometry

A total of 3 × 10^5^ SaOS-2 cells were seeded and incubated with Lonafarnib or Tipifarnib (20 µM) and osteogenic medium as indicated above. The cells were collected with a scraper and incubated with flow cytometry staining master mix or antibody master mix (Muse Annexin V and Dead Cell Kit, Merck Millipore) according to the manufacturer’s instructions to detect viable or apoptotic cells, respectively. The samples were read in duplicate on a flow cytometer) and 20000 events per sample were assayed.

### Nuclear fraction extraction

Nuclear fractions were extracted via a rapid mammalian nuclei isolation kit (CellLytic NuCLEAR Extraction Kit, Sigma-Aldrich). In brief, 2.5 × 10^6^ cells were treated with 10 µM or 20 µM Lonafarnib or Tipifarnib , respectively, and dissolved in fresh osteogenic medium. After treatment, the medium was discarded, and the cells were washed (2x DPBS) and harvested for consecutive lysis in hypotonic extraction buffer. The cytosolic and nuclear fractions were collected, shock-frozen and stored at −80 °C until further use.

### ALP activity assay

The relative activity of ALP was determined via a colourimetric *p-*nitrophenyl phosphate (*p*NPP) assay (AAT Bioquest, California, United States). In brief, whole-cell lysates were quantified via the Bradford assay (Bio-Rad Laboratories, Hercules, CA, USA); equal amounts of protein were incubated with 50 µl of *p*NPP as a substrate for 30 min in the dark at room temperature. The absorbance was measured at 400 nm via a multiplate Tecan reader. A bovine ALP standard was used to construct a standard curve and calculate the amount of ALP [mU/ml]. For analysis of direct effects of FTIs on ALP, a continuous fluorescence assay was performed. A total of 38 mU of bovine ALP standard was incubated with varying amounts of Lonafarnib and Tipifarnib (10–100 µM). A kinetic cycle was used for 30 min, with reading points every 30 s. After 2 min of background signal measurement, the assay was paused, and the reaction started by the addition of 2 µl of *p*NPP.

### Western blot

The cells were harvested with a cell scraper, briefly centrifuged, and then lysed with radioimmunoprecipitation (RIPA) buffer. The protein samples (20 µg/lane) were separated by sodium dodecyl sulphate-polyacrylamide gel electrophoresis (SDS-PAGE) and blotted onto a nitrocellulose membrane (Amersham, Protran, Sigma-Aldrich) via semidry blotting (Bio-Rad, 27 V, 30 min). The membrane was washed (1x TBS), blocked with bovine serum albumin blocking solution (LI-COR) and incubated with primary antibodies (anti-ALP, 1:1000, Abcam; anti-RUNX2, 1:400, Proteintech; anti-COL1A1, 1:200, Santa Cruz) for 1 h with shaking. For the loading control, we used an anti-β-actin antibody (LI-COR). After thorough washing (4 × 5 min in TBS + 0.1% Tween 20), the blots were incubated with secondary antibodies (goat anti-mouse, 1:10,000; goat anti-rabbit, 1:10,000; both LI-COR, 1 h at RT), washed (3 × 5 min in TBS + 0.1% Tween 20, 1 × 5 min in TBS), dried in the dark and visualised with an Odyssey reader (Odyssey CLx LI-COR).

### Isolation of extracellularly mineralizing vesicles

SaOS-2 cells were seeded at a density of 5 × 10^6^ on 150 mm TC dishes under the conditions mentioned above. The supernatants were depleted of cell debris via centrifugation (4000 rpm, 20 min; Optima L-90 K; Beckmann Coulter, Brea, CA, USA). Afterwards, the supernatant was transferred to thick-walled ultracentrifuge tubes (Beckmann Coulter) and subjected to ultracentrifugation (Optima L-90 K; Beckmann Coulter) utilizing the SW28 swing-out bucket rotor (Beckmann Coulter) at 150,000 × g at 4 °C for 90 min.

The pellet was resuspended in DPBS and then centrifuged again in a micro-ultracentrifuge (100,000 × g, 4 °C, 1 h; Discovery 90, Sorvall). Extracellular vesicles were analysed for size and amount via dynamic light scattering (Zetasizer Ultra, Malvern Panalytical, Ltd., Worcestershire, UK).

### EV characterisation by DLS

Particle size was determined by dynamic light scattering (DLS) with a Zetasizer Ultra (Malvern Panalytical, Malvern, UK) in a ZSU1002 Capillary Cell. A volume of 10 µl purified EV suspension was loaded to the capillary cell and measured with 15 repeats, at 25 °C (equilibration time: 120 s, pause between repeats: 10 s, scattering collection angle: 90°, all other parameters set to auto mode). Polydispersity Index was < 0.3 and Z-Average (mean of all particles) as well as the main peak (ordered by intensity) were between 170 and 300 nm for all samples.

Particle concentration was determined with the same device as particle size, using a glass cuvette with square aperture (PCS1115). Samples were diluted 1:20 in DPBS to a total volume of 1 ml. Particle concentration measurement was performed at 25 °C with a dispersant scattering mean count rate of 279 kcps (kilo counts per second), 120 s equilibration time, no pause between repeats and with the general purpose analysis model. Particle concentration range was about $\:5\times{10}^{7}-5\times{10}^{8}$ particles/ml for all samples.

### RNA extraction

With the Quick-RNA MiniPrep Kit (Zymo Research, Freiburg, Germany), complete RNA was extracted from SaOS-2 cells. In brief, 5 × 10^6^ cells were seeded and treated with the inhibitors Lonafarnib and Tipifarnib in osteogenic medium in the same way as described previously. A total of 600 µl of RNA lysis buffer was used to lyse the cells. After the cell layers were scraped with a cell scraper, the cells were transferred to a Spin-Away Filter column in a 1.5 ml Eppendorf tube and centrifuged. (10,000 × g, 30 s). The columns were discarded, and the supernatant was mixed with the same volume of ethanol. The solution was transferred to another column and centrifuged (10,000 × g, 30 s). The supernatant was discarded, and the Spin-Away Filter column was transferred to a fresh Eppendorf tube. Subsequently, 400 µl of RNA Prep Buffer was added, and the mixture was centrifuged again (10,000 × g for 30 s). The supernatant was discarded again, and 700 µl of RNA wash buffer was added to the Spin-Away Filter column, which was subsequently centrifuged (10,000 × g for 30 s). After the supernatant was discarded, 400 µl of RNA wash buffer was added to the column, which was subsequently centrifuged (10,000 × g for 60 s) to ensure complete removal of the wash buffer. The Spin-Away Filter column was then transferred to a new Eppendorf tube. Total RNA was eluted in 100 µl of RNase-free H_2_O.

### Library Preparation and nanopore sequencing

For transcriptomic analysis, the nanopore technique was used. For cDNA synthesis and library preparation, the cDNA-PCR Sequencing Kit V14 was used according to the manufacturer’s instructions (SQK-PCS114, Oxford Nanopore Technologies, Oxford, UK). RNA was extracted as stated above from SaOS-2 cells treated for 72 h with Lonafarnib (20 µM) and from untreated SaOS-2 cells. A total of 200 ng of RNA from treated and untreated samples was used in three biological replicates. The FLO-MIN106D Spot-On Flow Cell R9 Version was used, and the number of pores was tested before use. For sequencing, the samples were prepared with a Native Barcoding Kit 24 V14 (SQK-NBC111.24, Oxford Nanopore Technologies).

### MinION data analysis

The raw sequence data were basecalled in real time via the integrated Guppy software 7.1.4 of the MinION Mk1C. The Epi2me labs workflow wf-transcriptomes was used for alignment, differential gene expression analysis and differential transcript usage. Salmon was used to assign reads to individual annotated isoforms defined by the GTF-format annotation. These counts were used to perform statistical analysis to identify the genes and isoforms that showed differences in abundance between the experimental conditions. A statistical analysis was performed via edgeR to identify the subset of differentially expressed genes using the gene counts as input. The normalisation factor was calculated for each sequence via the default TMM method [42].

The differentially expressed genes were corrected for false discovery (FDR) using the method of Benjamini & Hochberg [43]. Differential transcript usage was assessed via the R package DEXSeq.

A heatmap was produced via R script heatmap2 running on a galaxy cloud-based server. The sequencing data were uploaded to the Galaxy web platform, and we used the public server at usegalaxy.org to analyse the data [44].

### GeneMANIA analysis

For the interaction network analysis “The GeneMANIA prediction server: biological network integration for gene priorization and predicting gene function” by Warde-Farley et al. was used. GeneMANIA predicts gene function by using the composite network using a variation of the Gaussian field label propagation algorithm.

### STRING network cluster analysis

For STRING analysis, we used the open free STRING network database. Local STRING network cluster or simply STRING clusters are precomputed clusters derived by hierarchically clustering the full STRING network using an average linkage algorithm. The names are derived automatically based on a cluster’s consensus protein annotations taken from GO, KEGG, Reactome, UniProt, SMART and InterPro.

### Proteome Analysis

For proteomic analysis of a specific protein band, a nanoHPLC-ESI-MS/MS analysis was performed by an external service provider. To this end, 2.5 x 10⁶ cells were seeded in 10 cm Petri dishes and incubated for 24 h under standard conditions. Subsequently, cells were treated with 10 ml of osteogenic medium supplemented with 10 µM Lonafarnib or Tipifarnib. After the indicated incubation period, cells were lysed as described in section 2.2.3, and protein concentrations were determined using the Bradford assay. Equal amounts of protein (30 µg per sample) were separated by SDS-PAGE. Specific bands from the Lonafarnib- and Tipifarnib-treated groups were excised using a sterile scalpel, transferred to 1.5 ml microcentrifuge tubes, and submitted for proteomic analysis. Proteins were separated by nanoscale high-performance liquid chromatography (nanoHPLC), ionised via electrospray ionisation (ESI), and analysed by tandem mass spectrometry (MS/MS).

### Statistics

Statistical analysis was performed with the software GraphPad Prism 9 (GraphPad Software, Inc., La Jolla, CA, United States). The data were evaluated via one-way ANOVA, and corrections for multiple comparisons were performed via Tukey’s *post hoc* test. All the experiments were repeated at least three times, and the data are reported as the means ± SDs. Statistical significance is indicated as follows: *p < 0.05; **p < 0.01; *** p < 0.001; **** p < 0.0001; *ns = non-significant*.

## Results

### Treatment with FTIs decreases the mineralization of SaOS-2 cells

**Fig. 1:**
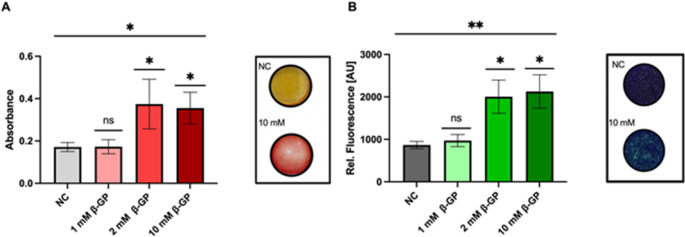
Mineralization ability of SaOS-2 cells after β-GP treatment. ARS staining (**A**) and tetracycline staining (**B**) of SaOS-2 cells stimulated with 50 µg/ml ascorbic acid and different concentrations of β-GP. NC = Negative control (unstimulated). Columns shown represent absorbance mean values (**A**) and fluorescence mean values (**B**) with SD of at least three independent replicates. Statistical significance is indicated as follows: * p < 0.05; ** p < 0.01

In this experimental study, we examined the molecular impact of the FTIs Lonafarnib and Tipifarnib on MVM in SaOS-2 cells. To determine their mineralization ability, we stimulated the cells by adding 1, 2 and 10 mM β-GP and 50 µg/ml ascorbic acid. ARS staining was subsequently used for the quantification of mineral deposition. The anthraquinone scaffold forms complexes with positively charged cations, and the absorbance can be measured. Compared with ARS staining, tetracycline staining also forms complexes with cations but exhibits a fluorescence signal upon stimulation, with no significant difference **(**Fig. 1**).** After treatment with 2 mM β-GP (*mean* = 0.3743, *p* = 0.0220, *SEM* = 0.0677, *lower 95% CI* = 0.0829, *upper 95% CI* = 0.6657) and after 10 mM β-GP (*mean* = 0.355, *p* = 0.0348, *SEM =* 0.0429, *95% CI* = 0.1706–0.5406; *ANOVA* summary (Fig. 1A): *p* = 0.018, F_3, 8_ = 1.055) we measured a significant increase in the absorbance signal equal to the mineral deposition with ARS staining and also for the tetracycline staining with 2 mM β-GP (*mean* = 2004, *p* = 0.0243; *SEM* = 391.0, *95% CI* = 1103–2906) and 10 mM β-GP (*mean* = 2127, *p* = 0.0116, *SEM* = 393.6, *95% CI* = 1220–3035; *ANOVA* (Fig. 1B): *p* = 0.0045, F_3, 32_ = 3.149).

**Fig. 2:**
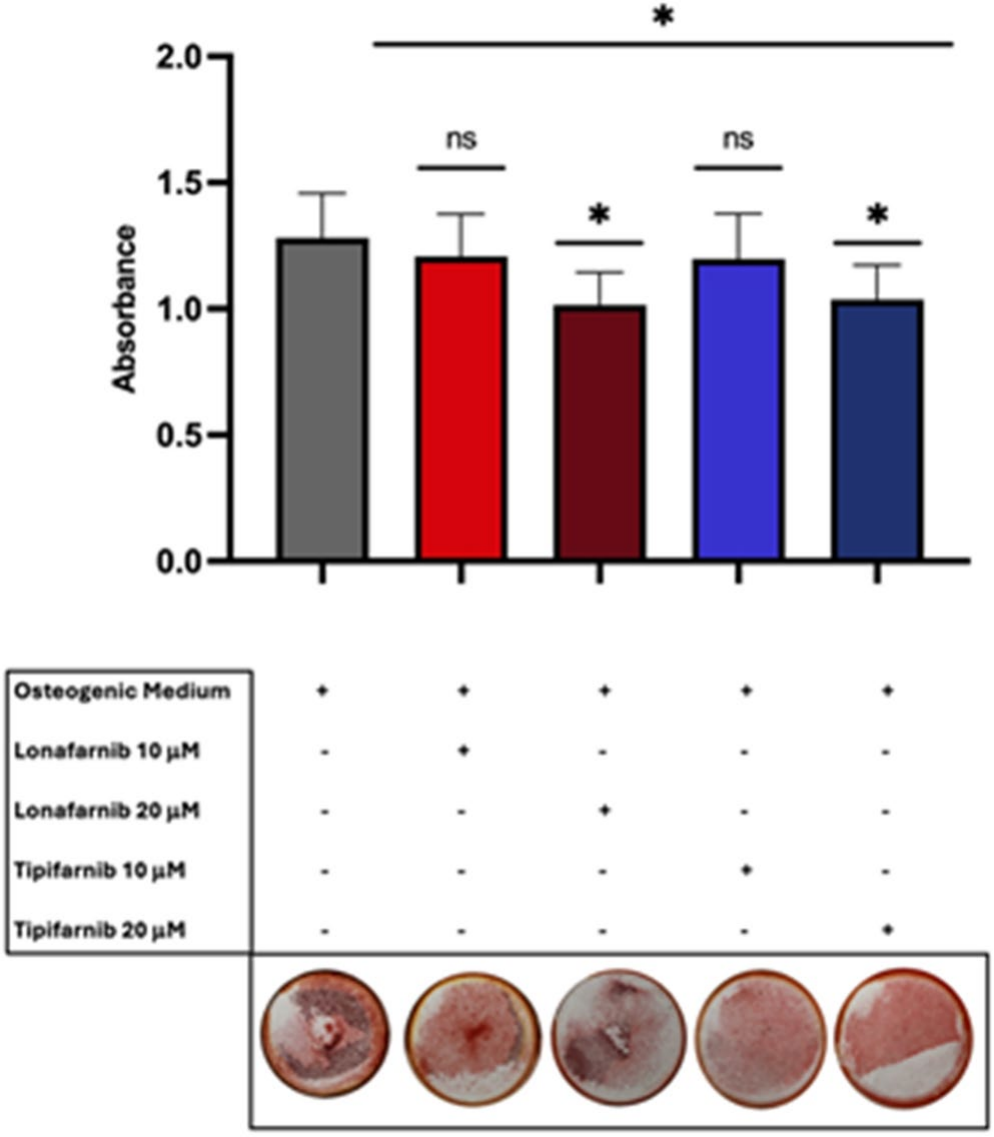
High FTI concentrations lead to decreased mineralization of SaOS-2 cells. Mineralization of SaOS-2 cells after treatment with Lonafarnib or Tipifarnib in osteogenic medium for 3 days. Alizarin images were taken in a 6-well plate with a camera. The darker the colour is, the stronger the mineralization. Columns shown represent absorbance mean values with SD of 3 independent replicates. Statistical significance is indicated as follows: ns = non-significant; * p < 0.05

**Fig. 3:**
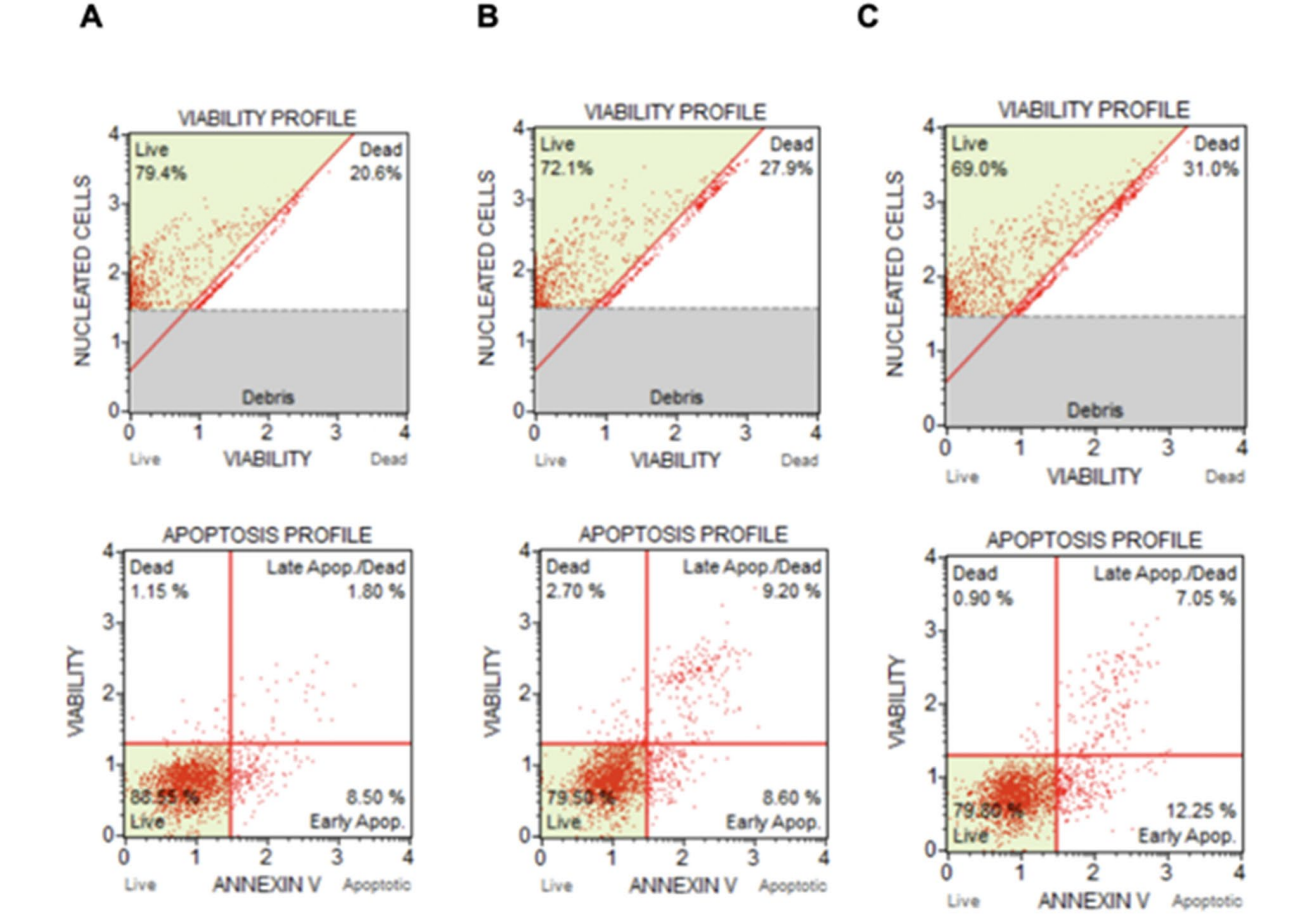
Viability and apoptosis analysis by flow cytometry. Flow cytometric viability assay (upper dot plot) and apoptosis assay with annexin V (lower dot plot) for cells treated with osteogenic medium for 72 h (**A**), with osteogenic medium supplemented with 20 µM Lonafarnib for 72 h (**B**) and with osteogenic medium supplemented with 20 µM Tipifarnib for 72 h (**C**)

After optimization of the mineralization conditions, the mineral deposition was examined via ARS staining after treatment for 3 days with 10 µM or 20 µM Lonafarnib or Tipifarnib, respectively. After treatment with 10 µM FTIs, mineralization was not significantly affected, whereas treatment with 20 µM Lonafarnib (*mean* = 1.014, *p* = 0.0271, *SD* = 0.1520, *SEM* = 0.0679, *95% CI* = 0.5752–1.9528) or Tipifarnib (*mean* = 0.8885, *p* = 0.0220, *SD* = 0.0956, *SEM* = 0.0429, *95% CI* = 0.7649–1.008; *ANOVA* **(2)** *p* = 0.0313, F_4, 25_ = 3.157) significantly decreased mineralization compared with that of the negative control, with little difference between the two inhibitors **(**Fig. 2**)**. To rule out a lethal effect of the inhibitors as a cause of the diminished mineralization, we performed flow cytometry on the treated cell samples. As shown in Fig. 3, the viability of the cells treated with 20 µM of each FTI was greater than that of the cells subjected to osteogenic differentiation, indicating that mineralization was in fact inhibited specifically and not a result of cell death.

### The accumulation of the mevalonate pathway intermediates FPP and GGPP does not affect mineralization

**Fig. 4:**
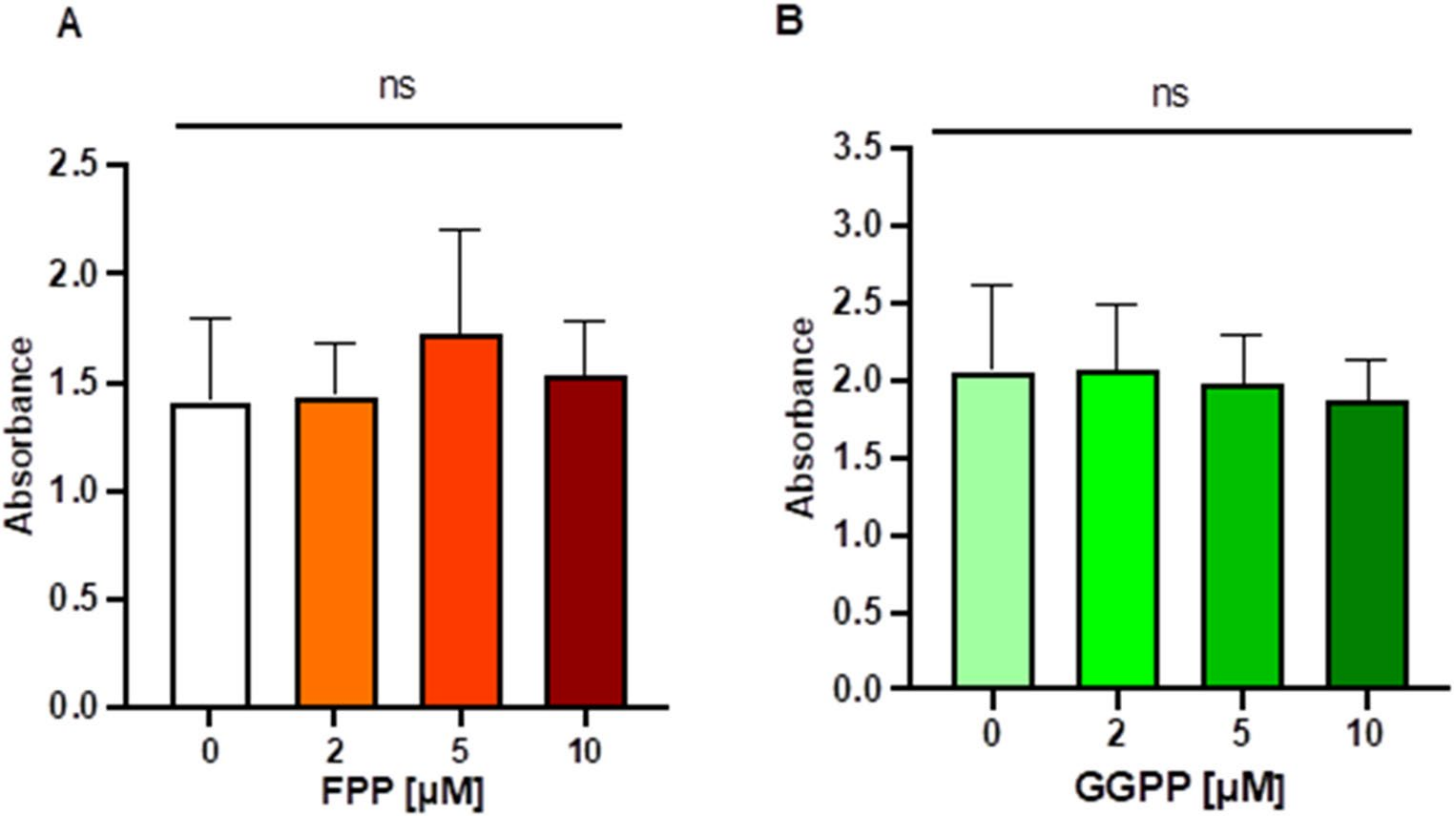
Effect of mevalonate pathway intermediates on MVM. ARS staining of SaOS-2 cells after treatment with different concentrations of the mevalonate pathway intermediates FPP (A) and GGPP (B) for 48 h in osteogenic differentiation medium. Columns shown represent absorbance mean values with SD of at least 3 independent replicates. Statistical significants is indicated as follows: ns = non-significant

To investigate why FTase inhibition leads to inhibited mineralization, we assessed several hypotheses. In general, mineralization is crucial for the formation of most hard eukaryotic tissues. Previous studies have indicated that the accumulation of FPP and its downstream molecule GGPP, intermediates of the mevalonate pathway, can inhibit the differentiation of mesenchymal stem cells into osteoblastic cells [28, 45]. Therefore, we hypothesized that FTase inhibition may lead to the accumulation of FPP/GGPP and thus decrease mineralization. SaOS-2 cells were treated for 48 h with exogenous FPP or GGPP at different concentrations 2 µM, 5 µM, or 10 µM). However, no significant decrease in mineralization was observed, as determined by ARS staining for FPP (*ANOVA p* = 0.2256, F_3, 32_ = 1.530 **(**Fig. 4A**)**) nor for GGPP (*ANOVA p* = 0.8525, F_3, 8_ = 0.2597 **(**Fig. 4B**)**). These findings suggest that the accumulation of these intermediates does not exert an inhibitory effect on mineralization in this context.

### The number of MVs significantly increases after Lonafarnib treatment

MVs are budded off membranes as described in numerous studies. Therefore, our second hypothesis was that the inhibition of FTase affects the physiological behaviour of these vesicles.

**Fig. 5:**
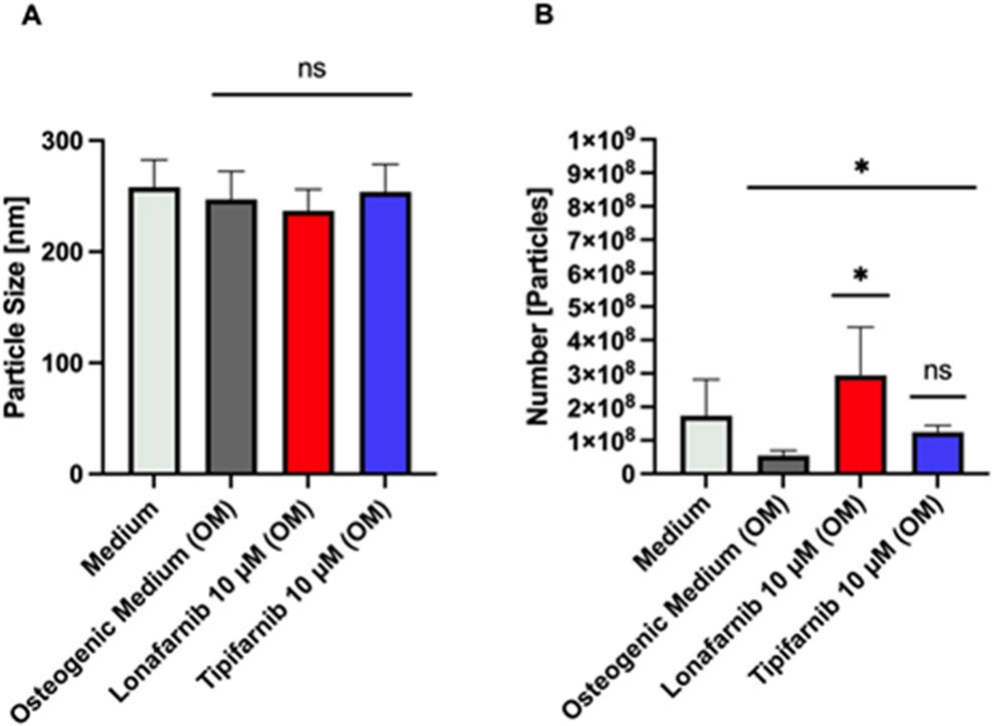
Effects of FTI treatment on the particle size and number of vesicles. Particle size (**A**) and number of vesicles (**B**) after the treatment of SaOS-2 cells with osteogenic medium supplemented with 10 µM Lonafarnib or Tipifarnib for 96 h. The supernatant was collected and ultracentrifuged. Columns shown represent particle size (**A**) and number of particles (**B**) mean values with SD of at least 3 independent replicates. Statistical significance is indicated as follows: ns = non-significant; * p < 0.05

We treated SaOS-2 cells as described, isolated small extracellular mineralizing vesicles via ultracentrifugation and analysed them via dynamic light scattering. The treatment did not affect the particle sizes, which ranged from 200 to 300 nm (Fig. 5A**)**. Surprisingly, we found a significant > fivefold increase in the number of supernatant vesicles after Lonafarnib treatment (*mean* = 2.94 x10^8^, *p* = 0.0230, *SD* = 1.44 x10^8^, *SEM* = 8.31 x10^7^, *95% CI* = −6.292 x10^7^ − 6.52 x10^8^; *ANOVA p* = 0.0321, F_3, 8_ = 6.437 **(**Fig. 5B**)**) compared with that after osteogenic medium treatment. The number of vesicles after Tipifarnib treatment increased 2.3-fold but did not significantly differ from that in the control group.

### FTIs exhibit no inhibitory effect on ALP function in a cell-free assay

**Fig. 6:**
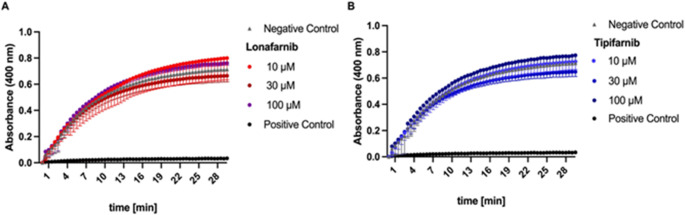
No effect on ALP activity after FTI treatment. The activity of ALP after FTI treatment is depicted. A standard amount of bovine ALP was incubated with different concentrations of Lonafarnib and Tipifarnib. The colourimetric change in the pNPP substrate was used to follow the ALP activity [mU/ml] in a continuous enzyme assay, and 0.1 M NaOH was used as a positive control

As FTI treatment led to an unexpected increase in the amount of MVs in the supernatant, we investigated whether FTIs exert inhibitory effects on ALP, the key enzyme involved in mineralization. To determine the change in ALP activity, we established a colourimetric cell-free enzyme assay by adding *p*NPP as a substrate to bovine ALP, which was incubated with Lonafarnib or Tipifarnib. As shown in Fig. 6, neither Lonafarnib nor Tipifarnib had a detectable effect on ALP activity.

### FTase Inhibition leads to decreased ALP and collagen type I expression by diminishing RUNX2 activity in SaOS-2 cells

**Fig. 7:**
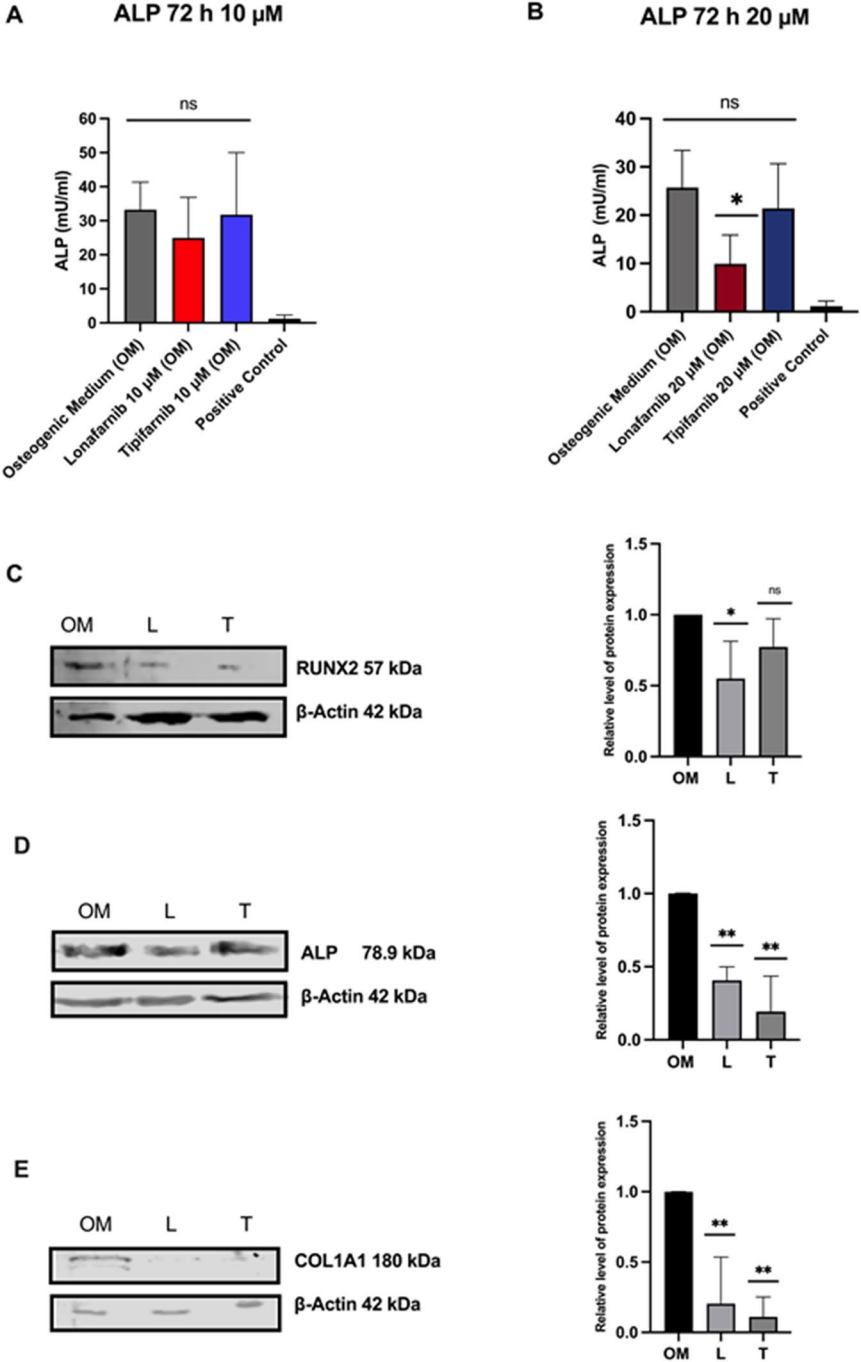
Effects of FTI treatment on ALP and collagen type I expression, as well as RUNX2 activity. ALP activity in SaOS-2 cells was measured after treatment with different concentrations of FTIs. The protein concentration of whole-cell lysates was determined via the Bradford assay, and the amount of ALP was detected via the pNPP assay (**A**, **B**). Columns shown represent ALP [mU/ml] mean values with SD of 3 independent replicates. Statistical significance is indicated as follows: ns = non-significant; * p < 0.05. Western blots of the proteins RUNX2 (**C**), ALP (**D**) and COL1A1 (**E**) after treatment for 72 h with 20 µM Lonafarnib or Tipifarnib in osteogenic medium is shown. Nucleus extraction was performed for RUNX2 detection, whole-cell lysates were isolated for ALP analysis, and the supernatants were collected for Western blot analysis of COL1A1. The protein concentration was quantified via the Bradford assay. β-Actin was used as the loading control. The following primary and secondary antibodies were used: anti-ALP, 1:1000, ab126820, Abcam; anti-RUNX2, 1:400, Proteintech; anti-COL1A1, 1:200, Santa Cruz; anti-β-actin antibody (D11129-01, LI-COR), goat anti-mouse, 1:10,000, C70301-03; goat anti-rabbit, 1:10,000, 925–68071, both LI-COR; OM = osteogenic medium, L = Lonafarnib 20 µM, T = Tipifarnib 20 µM, M = cell medium. ImageJ was used for Western blot quantification. Mean intensity normalised against the control intensity of three independent samples is shown. Uncropped western blots of RUNX2 (Fig. S1), ALP (Fig. S2) and COL1A1 (Fig. S3) are shown at supplementary information. Mean intensity normalized against the control intensity of three independent samples is shown. Uncropped western blots of RUNX2 (Fig. S1), ALP (Fig. S2) and COL1A1 (Fig. S3) are shown at supplementary information. Statistical significance is indicated as follows: ns = non-significant; * p < 0.05; ** p < 0.01

Endogenous ALP activity was tested after Lonafarnib and Tipifarnib treatment. Therefore, the cells were treated with 10 µM or 20 µM of the inhibitors for 72 h. The same protein amounts quantified with the Bradford assay were used and incubated with *p*NPP for 20 min (Fig. 7A). ALP mean activity decreased from 33.26 [mU/ml] to 24.95 [mU/ml] for 10 µM Lonafarnib and to 31.77 [mU/ml] for 10 µM Tipifarnib with no statistical significance. After 20 µM treatment, the activity decreased significantly (from 37.68 [mU/ml] to 14.90 [mU/ml] for Lonafarnib **(***p* = 0.0474, *SD* = 8.518, *SEM* = 4.918, *95% CI* = −6.260–36.06) and to 32.05 [mU/ml] for Tipifarnib failing to reach statistical significance (Fig. 7B**)**. Although both treatments did not yield statistically significant results, a clear trend for the Lonafarnib treatment was observed, which was further confirmed by Western blot analysis. The blots revealed a weaker band at approximately 78.9 kDa, especially after Lonafarnib treatment (Fig. 7D), suggesting reduced expression. Moreover, we found decreased expression of COL1A1, a protein that mostly defines the ECM and is crucial for mineralization in the samples treated with FTIs. (Fig. 7E**)**

To gain deeper insight into this molecular pathway, we conducted Western blot analysis of RUNX2, which is known to be the major transcription factor involved in osteogenic differentiation and crucial for the expression of ALP [46, 47]. The Western blots revealed a distinct band of RUNX2 in the control group (osteogenic medium) compared with the treatment group, which lacked mineralization and consequently contained lower amounts of ALP to orchestrate MVM (Fig. 7D**).**

### Nanopore transcriptome sequencing reveals downregulation of the RUNX2 and ALP transcripts

**Fig. 8:**
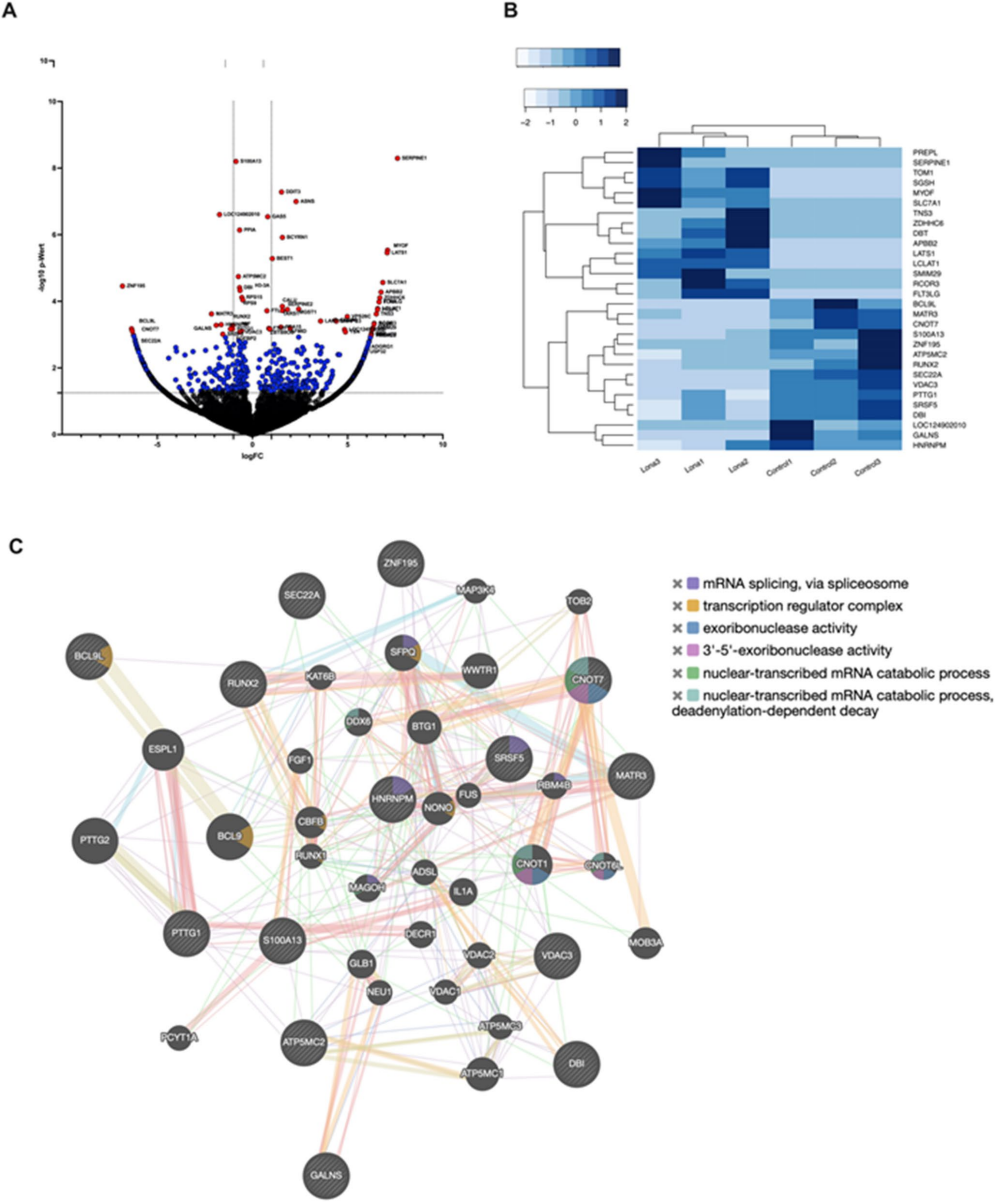
Results of DGE after Lonafarnib treatment. SaOS-2 cells were treated with 20 µM Lonafarnib for 72 h, and the transcriptome was analysed and compared with that of the control group. The results of DGE and DTU are shown. Volcano plot (**A**) showing the results of the DGE. The logFC values are plotted against the -log10 p values, and the red dots are significant genes with p values < 0.05 and FDRs < 0.2. The blue dots represent non-significant genes with p values < 0.05 and FDRs > 0.2. The black dots represent non-significant genes with p values > 0.05 and FDRs > 0.2 Heatmap (**B**) shows the z scores of the 15 top significantly up- and downregulated genes of DGE. A gene network created with GeneMANIA shows the 15 most downregulated genes. (**C**) Striped Circles indicate genes detected by DTU. Red connection lines show physical interactions of the genes, purple connection lines show predicted co-expression and green the genetic interactions of the genes

As Lonafarnib treatment resulted in a significant decrease in ALP expression and because of its clinical usage in Progeria, we decided to perform the transcriptome analysis of the RNA from Lonafarnib treated SaOS-2 cells. A total of 1217 nanopores were available on the flow cell according to the control software prior to sequencing. After 72 h of sequencing, 2,970,000 reads were successfully mapped against the human reference genome GRCh38. Figure 8A shows the result of the differential gene expression (DGE) depicted as a Volcano plot [48]. The logFC values are plotted against the -log10 *p*-values, and the red dots are significant genes with p values < 0.05 and FDRs < 0.2. Out of these significant red dots the 15 genes whose expression were most strongly upregulated and the 15 genes whose expression were most strongly downregulated in DGE are summarized as a heatmap. With the Euclidean distance method, a gene cluster was created to visualise similarities in the expression patterns (Fig. 8B).

**Fig. 9:**
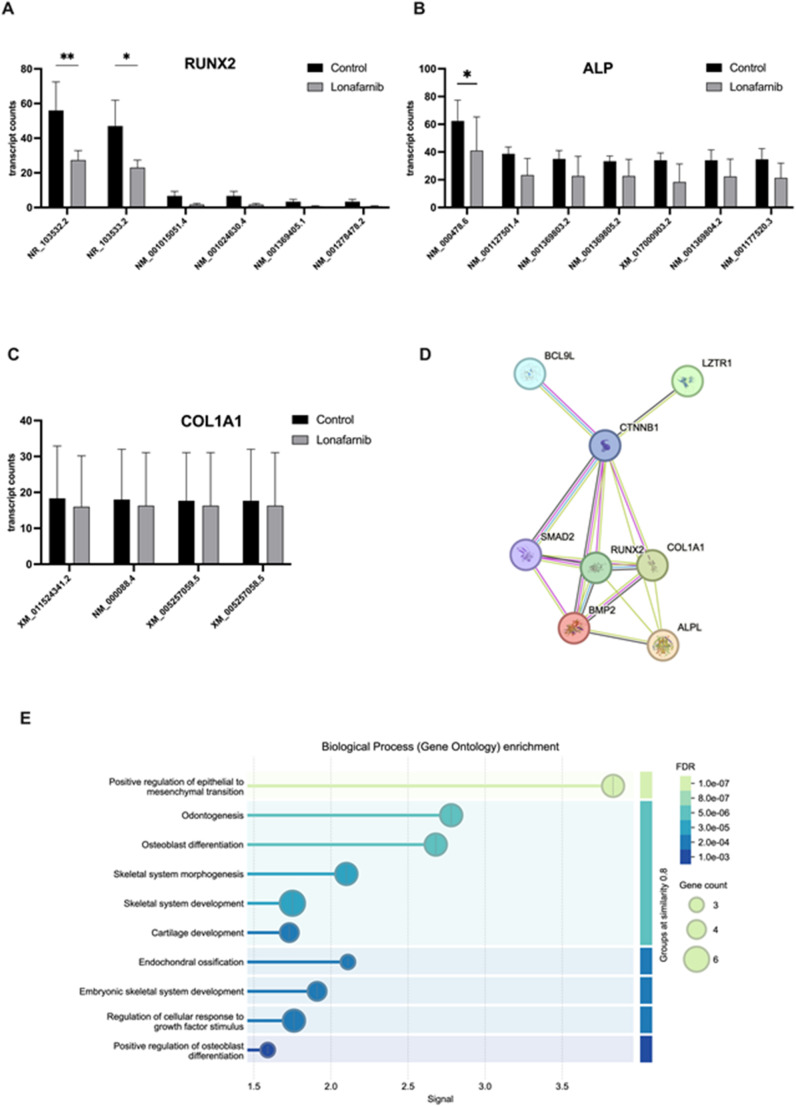
Results of DTU after Lonafarnib treatment. SaOS-2 cells were treated with 20 µM Lonafarnib for 72 h, and the transcriptome was analysed and compared with that of the control group. The results of DTU are shown. The transcript variant counts of the osteogenic markers RUNX2 **(A)**, ALP **(B)** and COL1A1 **(C)** were analysed and plotted. Columns plotted show mean transcript count with SEM of three independent samples. The STRING network cluster **(D)** shows the precomputed functional protein association network of the osteogenic genes and their gene neighbourhood. A Gene Ontology enrichment analysis was performed and shows biological processes associated with the target genes **(E). **Statistical significance is indicated as follows: * *p* < 0.05; ** *p* < 0.01

A GeneMANIA analysis was performed to show molecular functions of the downregulated genes. Six different transcript variants were detected in the RUNX2 gene. The number of all counts per million reads (CPM) was reduced after treatment with Lonafarnib. The RNA sequence NR_103532.2 was transcribed, with an average of 56 copies in the control group and 27 copies in the treatment group (*mean* = 27.33, *p* = 0.0069, *95% CI* = 8.656–48.68) (Fig. 9A). The CPM of the RNA sequence NR_103533.2 also decreased from 47 to 23 (*mean* = 23.00, *p* = 0.0208, *95% CI* = 3.990–44.01) For ALP, seven alternatively spliced transcripts were detected. NM_000478.6 (tissue-nonspecific isozyme isoform 1 preprotein) was detected in the control group, with an average of 62.33 CPM. In the treatment group, the number was reduced to 41 (*mean* = 41.00, *p* = 0.0357, *95% CI* = −19.29–101.3) (Fig. 9B). In contrast to the Western blot analysis results, no significant changes in the four COL1A1 transcript variants were detected (Fig. 9C).

### FTI treatment induces distinct protein signatures in a 70 kDa band

**Fig. 10:**
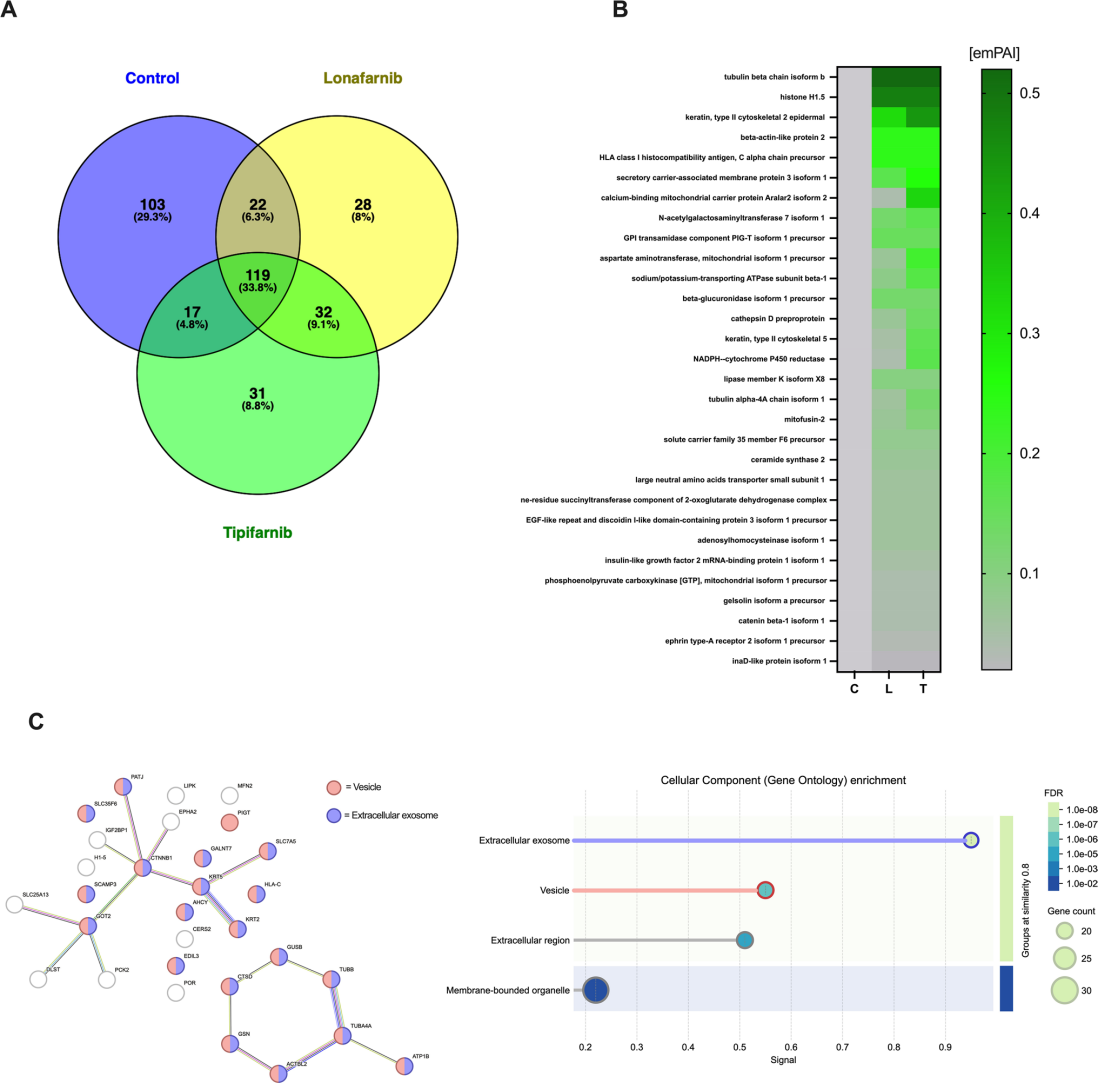
Proteomic analysis of a ~70 kDa band from SaOS-2 cells treated with farnesyltransferaseinhibitors Lonafarnib and Tipifarnib (**A**) Venn diagram showing the number and percentage ofproteins detected in control, Lonafarnib-, and Tipifarnib-treated groups, including shared andgroup-specific proteins. (**B**) Heatmap of emPAI values for selected proteins exclusively detectedin the Lonafarnib and Tipifarnib groups. Green indicates relative protein abundance; greyindicates absence in the control group. C = Control, L = Lonafarnib, T = Tipifarnib (**C**) Protein–protein interaction network [49] and Gene Ontology (GO) enrichment analysis for the category"Cellular Component" (right) of differentially abundant proteins. Key enriched terms includeextracellular exosome, vesicle, and membrane-bounded organelle.

In SaOS-2 cells treated with the farnesyltransferase inhibitors Lonafarnib or Tipifarnib, a prominent protein band around 70 kDa consistently appeared on SDS-PAGE gels. This band was absent in both the osteogenic medium (OM) control and the medium-only (M) group, suggesting an FTI-specific effect (Fig. S2). The band was excised and analysed by nanoHPLC-ESI-MS/MS. Mass spectrometry identified 260 proteins in the OM group, 200 in the Lonafarnib group, and 203 in the Tipifarnib group. Of these, 60 proteins were uniquely detected following Lonafarnib treatment and 63 after Tipifarnib. A set of 118 proteins was found across all three conditions (Fig. 10A). Full protein lists are available in Tables S1–S7. Fig. 10B shows a heatmap of proteins exclusively detected in FTI-treated cells, regardless of their molecular weight. To specifically assess proteins within the excised band, those detected in the 66–72 kDa range are shown separately in Fig. S3. Among the proteins uniquely identified in the FTI-treated groups, CTNNB1 (β-Catenin) stood out as the only factor with a known indirect link to mineralization, primarily via Wnt signalling [50]. 

## Discussion

In this study, we explored the role of farnesylation in MVM. Our findings revealed that the inhibitors Lonafarnib and Tipifarnib reduced RUNX2-mediated ALP and COL1A1 expression, consequently decreasing mineralization (Fig. 7, 8 and 9).

The exploration of farnesylation and the potential inhibition of this process via FTIs shows promise for treating a range of diseases, such as cancer, hepatitis D, and progeria [48, 50, 51]. However, to our knowledge, this is the first study describing the inhibitory effect of FTIs on MVM in SaOS-2 osteosarcoma cells. We observed a reduction in mineralization following treatment with Lonafarnib and Tipifarnib, as indicated by ARS staining.

FPP is the substrate of farnesyltransferase. This hydrophobic isoprenoid is produced in the well-studied mevalonate pathway, a finely tuned critical metabolic pathway involved in various essential cellular functions, such as biosynthesis of the cholesterol precursor scaffold of steroid hormones [52–55]. We hypothesized that the molecular inhibition of FTase results in the accumulation of FPP or its downstream molecule GGPP, thereby inhibiting mineralization. Weivoda and Hohl reported that the inhibition of squalene synthase, an enzyme that catalyses the formation of squalene from FPP, by zaragozic acid leads to increased levels of endogenous FPP and GGPP, which nearly prevented matrix mineralization in their osteoblast model [45, 56]. Furthermore, these researchers tested exogenous doses of FPP ranging from 1 to 10 µM, which led to a significant decrease in calvarial osteoblast mineralization. Surprisingly, the application of exogenous FPP and GGPP did not lead to reduced mineralization in our experimental model. This observation could be attributed to the peculiar nature of SaOS-2 cells, which are immortalized osteosarcoma cells that exhibit major alterations in their cellular physiology. Therefore, it cannot be ruled out that these negatively charged molecules are unable to pass through the SaOS-2 cell membrane.

Moreover, the quantity of FPP utilized for protein prenylation may be considerably lower than the amount required for cholesterol synthesis. Furthermore, increased intracellular cholesterol levels are ubiquitously present in cancer cells, suggesting that increased amounts of FPP are converted by squalene synthase [57]. These findings suggest that the inhibition of FTase may lead to non-significant changes in the intracellular FPP levels.

To identify another explanation for the FTI-mediated amelioration of matrix mineralization, we determined the amount of mineralized MVs. Our study revealed that the number of MVs, budding off from osteogenic cells, is markedly increased in the supernatant following osteogenic stimulation and treatment with FTIs [58]. To our knowledge, this is the first report of changing MV levels after treatment with FTIs. However, the term “matrix vesicle” more commonly refers to mineralizing particles found within the ECM, but their presence is difficult to distinguish from that of nonmineralizing extracellular vesicles in the supernatant. Therefore, we cannot determine whether FTIs influence absolute vesicle quantity since we did not perform collagenase treatment before purification. MVs are secreted mainly by hard tissue-forming cells into the ECM and contain many enzymes, lipids, and organic compounds such as phosphates and calcium [59]. The interplay between the collagen-rich ECM and MVs triggers the formation of hydroxyapatite crystals, fostering the mineralization process of hard tissues [60]. Within this frame of reference, MVs may accumulate in the supernatant because of insufficient amounts of ECM, expressed by the diminished amounts of collagen type 1 we observed in this study. The vesicles cannot undergo physiological mineralization and remain in their natural shape, detectable by dynamic light scattering, but are unable to orchestrate MVM. In this regard, quantifying only the particles from the supernatant appears to be a less common but viable approach. Lonafarnib significantly increased the number of secreted vesicles in contrast to Tipifarnib, which showed a similar tendency but seemed to be less potent (Fig. 5B). Although both molecules are nonpeptidomimetics, this effect may be a consequence of pharmacokinetic differences related to their chemical scaffolds. This finding was also shown by Weber et al., who described a less potent effect of Tipifarnib than Lonafarnib on bacterial load reduction in a time-kill assay [51].

Since their initial discovery in 1967, numerous studies have consistently highlighted the distinctive nature of MVs as extracellular membrane-bound microparticles, which play crucial roles in the initial stages of mineral formation in bone and serve as the foundation for apatite generation in various other mineralizing tissues found in vertebrates. ALP is ubiquitously expressed in mammals and can be divided into four isoenzymes depending on its location of expression. One of the four ectoenzymes, tissue-nonspecific ALP, is expressed mainly in osteoblasts and chondrocytes, where it hydrolyses the main inhibitor of hydroxyapatite formation, pyrophosphate, to provide monomeric phosphate for the primary calcification inside the vesicles [58, 61]. Li et al. described the correlation between decreased ALP activity and reduced mineral deposition in SaOS-2 cells [62]. In the present study, we found that the inhibition of MVM by FTIs is accompanied by a reduction in ALP expression. Moreover, putative off-target effects on ALP function were not observed, as we were able to perform a cell-free enzyme assay (Fig. 6). There was a significant decrease in gene expression after treatment with 20 µM FTIs. Furthermore, the findings were confirmed by Western blotting. Interestingly, Lonafarnib again appeared to be more potent in this regard than Tipifarnib was, which could again be due to their varying chemical scaffolds.

Previously thought to be a passive structure, the collagen-rich ECM is essential for MVM. Type I collagen, which is composed of collagen type I alpha (COL1A1) and collagen type I alpha 2 (COL1A2), is a major organic component of bone [63, 64]. We found a decrease in COL1A1 protein expression after Lonafarnib treatment but no change in the expression of COL1A1 mRNA. However, there is no correlation between the abundance of protein and mRNA [65]. As our study revealed decreased collagen type-I and ALP expression, we investigated the activity of RUNX2, a transcription factor presumably involved in ALP and COL1A1 expression. RUNX2 can activate ALP expression in a nuclear matrix-targeting-dependent manner [66]. Also known as core-binding factor subunit alpha-1 (Cbfa1), RUNX2 belongs to the Runx superfamily and consists of RUNX1, RUNX2, and RUNX3. RUNX2 is encoded by two distinct promoters, P1 and P2, and is most frequently expressed in osteoblasts and chondrocytes. The transcription factor heterodimerizes with the core-binding factor-β (Cbfb), enabling DNA binding ability and stability [67–69]. The relevance of RUNX2 for biomineralization is evident, as it constitutes the key transcription factor for the expression of major bone matrix protein genes, including *COL1A1* and *ALP* [47, 69, 70]. Moreover, RUNX2 is responsible for the commitment of mesenchymal stem cells to the osteoblast lineage [69]. Thus, mice lacking the *RUNX2* gene (*RUNX2*-/-) exhibit a deficiency in osteoblasts and impaired bone formation [69, 71–74]. Additionally, in humans, *RUNX2* gene mutations are associated with cleidocranial dysplasia syndrome (CCD), and Lin et al. reported the inhibition of osteoblastic differentiation in smooth vascular cells following the deletion of *RUNX2* [75–77]. Moreover, RUNX2 has been reported to be an important regulator of ectopic mineralization in PXE and ossification of the thoracic ligamentum [78, 79].

These findings highlight the pharmacological implications of RUNX2 in diseases associated with mineralization and underscore its potential as a therapeutic target. Our study revealed reduced *RUNX2* gene expression and translocation into the nucleus after Lonafarnib and Tipifarnib treatment compared with those in the control group. In contrast, the cells without stimulation for mineralization exhibited translocation of RUNX2 in the cytosolic fraction (Fig. 7C). These findings suggest that the inhibition of farnesyltransferase interrupts the translocation of RUNX2 into the nucleus, consequently impairing its activation and leading to deficient mineralization. However, RUNX2 itself does not carry a CAAX motif and can therefore neither be farnesylated nor be a putative target of FTI-related inhibition of mineralization. Nevertheless, the RUNX2 transcription factor has been reported to be phosphorylated and activated by the MAPK pathway [80–82], which depends on the farnesylation of Ras [83].

This pathway is stimulated either by the binding of integrins to COL1A1 or by osteogenic growth factors such as bone morphogenetic proteins and fibroblast growth factor-2 [81, 84]. Additionally, Langenbach et al. described the induction of osteogenesis in bone marrow stromal cells after dexamethasone treatment through the phosphorylation of RUNX2 via MAPK [47]. Furthermore, the use of U0126, a selective ERK1/2 inhibitor, completely blocked RUNX2 activation, and Kanno et al. reported that mechanically induced RUNX2 activation was inhibited by Ras depletion, further indicating that the observed effects of FTIs may be due to the inhibition of the Ras-MAPK-RUNX2 axis [85]. Given the observed reduction in RUNX2 expression, we also explored whether compensatory or regulatory pathways might be involved in the cellular response to FTI treatment. We therefore examined our transcriptomic dataset for markers associated with cell fate decisions and alternative differentiation programs, including SOX9, PPARγ, p21/CDKN1A, Cyclin D1, PCNA, and TGF-β1. Although some of these transcripts were detectable, their expression levels remained low and did not reach the significance thresholds required for reliable interpretation. To support this hypothesis, we performed an upstream regulatory analysis based on our transcriptomic data, including a STRING network analysis. This revealed a predicted inhibition of MAPK1 and MAPK3, two key kinases in the MAPK/ERK pathway known to promote RUNX2 activation and osteogenic gene expression (Fig. S1). Several studies have reported that Ras-MAPK promotes osteogenic gene expression, ultimately leading to the differentiation of progenitor cells towards the osteoblastic cell lineage [86–88]. In this context, Xiao et al. reported that MAPK signalling stimulates osteogenesis through phosphorylation and activation of the transcription factor RUNX2, which further supports our proposed mechanism of action of FTIs on mineralization [89]. Finally, Murtada et al. reported that treatment with Lonafarnib in a mouse model of Hutchinson–Gilford progeria syndrome alleviated vascular stiffness, and a trend towards lower mural calcification was reported, which could have been mediated by the reduced RUNX2 activation observed in our study [90].

Furthermore, the DTU revealed that the transcripts NR_103532.2 and NR_013533.2 were transcribed at a statistically significantly reduced level after Lonafarnib treatment. These two long-non-coding RNA transcript variants (lncRNA) lack essential parts of the coding region. There is currently no data on which RUNX2 transcripts are primarily expressed physiologically, what proportion of the detected non-coding transcripts represent and what their exact function are. Interestingly, downregulated genes like *CNOT7*,* SRSF5* and *HNRNPM* are involved in mRNA splicing via spliceosome. In addition, *CNOT7* seems to play a role in nuclear-transcribed mRNA catabolic processes and has exoribonuclease activity [90, 91].

Future research could take a closer look at the transcript variants and examine the transcript distribution in the physiological setting. There could possibly be a connection between the malignancy of the tumour cells, the mineralization and the expression of the RUNX2 transcript variants.

However, Ras is not the only protein requiring farnesylation. There are approximately 200 proteins that are farnesylated in humans [7]; therefore, it appears reasonable to suggest that other farnesylated proteins with canonical functions in mineralization may be impaired upon FTI treatment. In addition to the MAPK pathway, multiple other pathways, such as the bone morphogenetic protein pathway or the Wnt/β-catenin pathway, which is known to activate RUNX2 during osteogenic lineage commitment, seem to be involved in the expression and activation of RUNX2. Bone morphogenetic protein receptors are bound at the cell surface by ligands. Therefore, after receptor activation on the surface, the canonical Smad pathway regulates the transcription of target genes such as *RUNX2* in the nucleus [93, 99]. In this context, further investigation of the human farnesyl proteome is warranted. To complement the transcriptomic data, we performed proteomic profiling of a specific protein band exclusively detected in the FTI-treated groups. None of the proteins uniquely detected were directly associated with mineralization. The only protein with an indirect connection to mineralization was CTNNB1, encoding β-catenin, a central component of the canonical Wnt pathway and a mediator of cell–cell adhesion [97, 98]. Although detected in the proteomic analysis, its emPAI score was low (0.04) and did not account for the observed protein band. While no direct link to mineralization was found, β-catenin may still contribute via upstream effects on RUNX2, as described in other osteosarcoma models [100]. However, in SaOS-2 cells, this relationship appears to be inverse, and our study did not distinguish between active and inactive β-catenin forms — an aspect that warrants further investigation using phospho-specific antibodies.

**Fig. 11:**
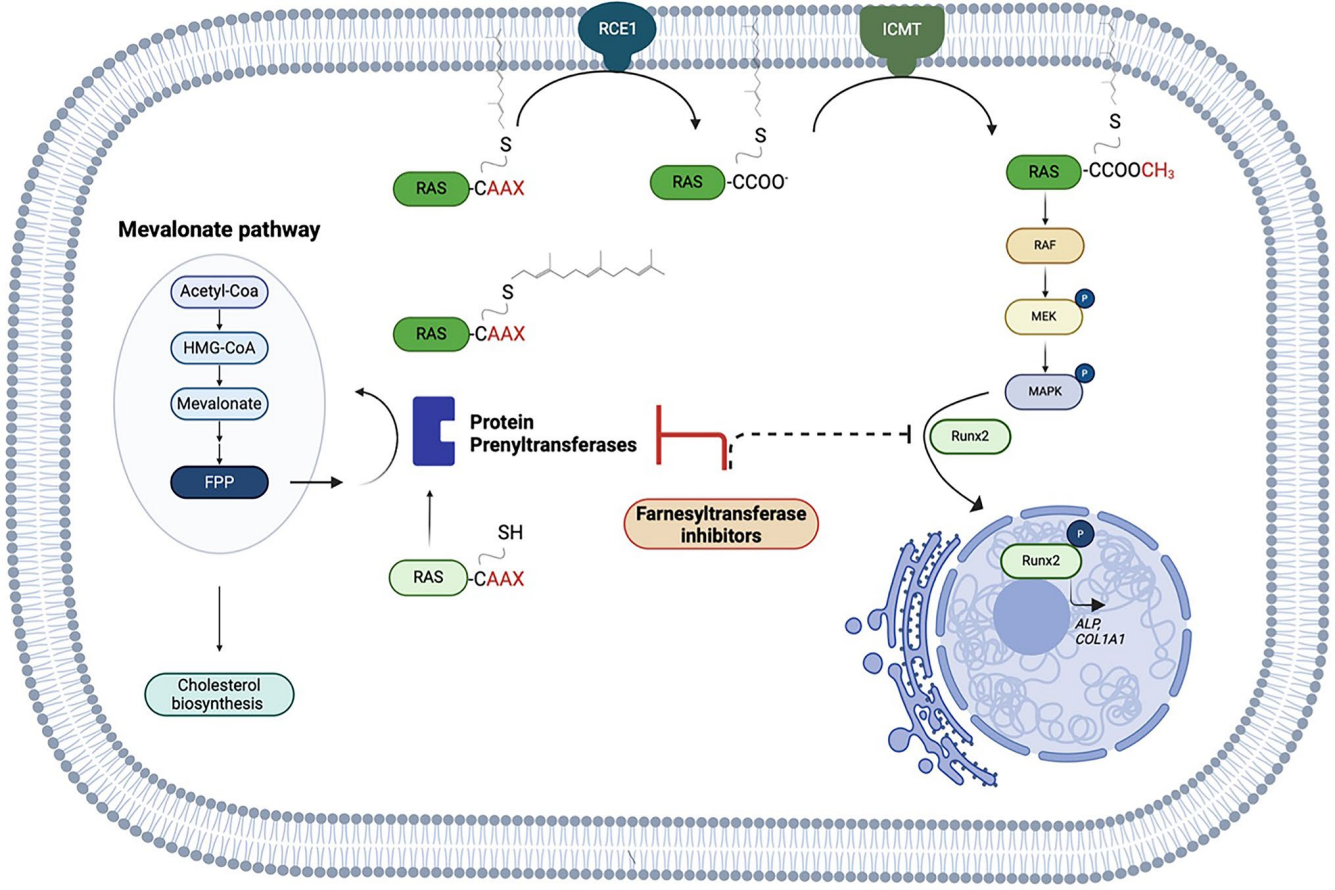
Interplay of the pathways putatively contributing to FTI-induced decrease in matrix-vesicle mediated mineralization in SaOS-2 cells. Experimental data presented in this study leading to a decrease in MVM. As described in the Results section, the inhibition of Ras and therefore the Raf/MEK/MAPK pathway plays a role in the phosphorylation of Runx2, therefore influencing ALP and Col1a1 expression

While our study demonstrates that FTIs significantly affect matrix-vesicle-mediated mineralization in SaOS-2 cells, we recognize that the SaOS-2 cell line, derived from osteosarcoma, may not fully mimic the complex architecture of normal bone tissue. However, as supported by Pautke et al. (2004), Strzelecka-Kiliszek et al. (2017), and Cutarelli et al. (2016), SaOS-2 cells remain a widely accepted pharmacological model for studying osteogenic endpoints [94–96]. Our methods, including ARS staining, ALP assays, and dynamic light scattering for vesicle quantification, have been validated in previous studies to reliably assess mineralization. Moreover, Duque et al. [41] highlight the regulatory role of protein isoprenylation in osteogenic differentiation, reinforcing our focus on farnesyltransferase inhibition [49]. Thus, while a more extensive characterisation of the extracellular matrix and vesicle populations would be required for a comprehensive bone biology study, our data robustly support the pharmacological effects of FTIs on mineralization processes. Overall, we were able to show for the first time that inhibiting farnesylation decreases RUNX2 activity in SaOS-2 cells, resulting in decreased MVM and decreased expression of ALP and COL1A1 in vitro (Fig. 11). Therefore, we elucidated the role of farnesylation in biomineralization. These findings provide new insights into the positive effects of statins and bisphosphonates on ectopic mineralization. Moreover, future research should explore the potential therapeutic use of farnesyltransferase inhibitors in addressing diseases associated with abnormal mineralization. Further studies are necessary to investigate the physiological role of farnesyltransferase, which will likely lead to a better understanding of the mechanisms and causes leading to pathologic mineralization.

In summary, we have demonstrated that the farnesyltransferase inhibitors Lonafarnib and Tipifarnib are able to interrupt MVM and that farnesylation might play a pivotal role in MVM, which needs to be investigated in further studies, as mineralization disorders pose considerable health risks in developed countries.

## Supplementary Information

Below is the link to the electronic supplementary material.

Supplementary Material 1

## Data Availability

The data supporting the findings of this study are available from the corresponding author upon reasonable request. Additional datasets generated and analysed during the study are included in the supplementary materials.

## Abbreviations

ALP: Alkaline Phosphatase.
ARS: Alizarin Red S.
ATCC: American Type Culture Collection.
β-GP: Beta-Glycerophosphate.
CAAX: Cysteine–Aliphatic–Aliphatic–X (motif).
CCD: Cleidocranial Dysplasia.
cDNA: Complementary DNA.
COL1A1: Collagen Type I Alpha 1.
CPM: Counts Per Million reads.
DMEM: Dulbecco’s Modified Eagle’s Medium.
DGE: Differential Gene Expression.
DMSO: Dimethyl Sulfoxide.
DPBS: Dulbecco’s Phosphate-Buffered Saline.
ECM: Extracellular Matrix.
ERK1/2: Extracellular Signal-Regulated Kinase 1/2.
FDPS: Farnesyl Diphosphate Synthase.
FDR: False Discovery Rate
FPP: Farnesyl Pyrophosphate.
FTase: Farnesyltransferase.
FTI(s): Farnesyltransferase Inhibitor(s)
FBS: Foetal Bovine Serum.
GGPP: Geranylgeranylpyrophosphate.
GGTase: Geranylgeranyltransferase (I, II, or III).
GRCh38: Genome Reference Consortium Human Build 38.
MAPK: Mitogen-Activated Protein Kinase.
MTT: 3-(4,5-Dimethylthiazol-2-yl)−2,5-Diphenyltetrazolium Bromide.
MVM: Matrix-Vesicle-Mediated Mineralization.
MVs: Matrix Vesicles.
NC: Negative Control.
OM: Osteogenic Medium.
PBS: Phosphate-Buffered Saline.
pNPP: p-Nitrophenyl Phosphate.
PCR: Polymerase Chain Reaction.
PXE: Pseudoxanthoma Elasticum.
Ras: Rat Sarcoma (small GTPase).
RIPA: Radioimmunoprecipitation Assay.
RUNX2: Runt-Related Transcription Factor 2.
Cbfa1: Core-binding factor subunit alpha-1 (alternate name for RUNX2).
Cbfb: Core-binding factor beta.
SDS-PAGE: Sodium Dodecyl Sulphate–Polyacrylamide Gel Electrophoresis.
SaOS-2: Human Osteosarcoma Cell Line.
SMAD: Small Mothers Against Decapentaplegic.
TBS: Tris-Buffered Saline.
TGF-β: Transforming Growth Factor-beta.
Wnt: Wingless/Integrated (signalling pathway).
