# Supplementary material for: Farnesyltransferase inhibitors decrease matrix-vesicle-mediated mineralization in SaOS-2 cells
Source: Mol Biol Rep. 2025 Nov 19;53(1):99. doi: 10.1007/s11033-025-11138-2 (PMC12630319; doi:10.1007/s11033-025-11138-2)
Supplement: Supplementary file 1 — Supplementary Material 1 [file 11033_2025_11138_MOESM1_ESM.docx]

**Supplementary**


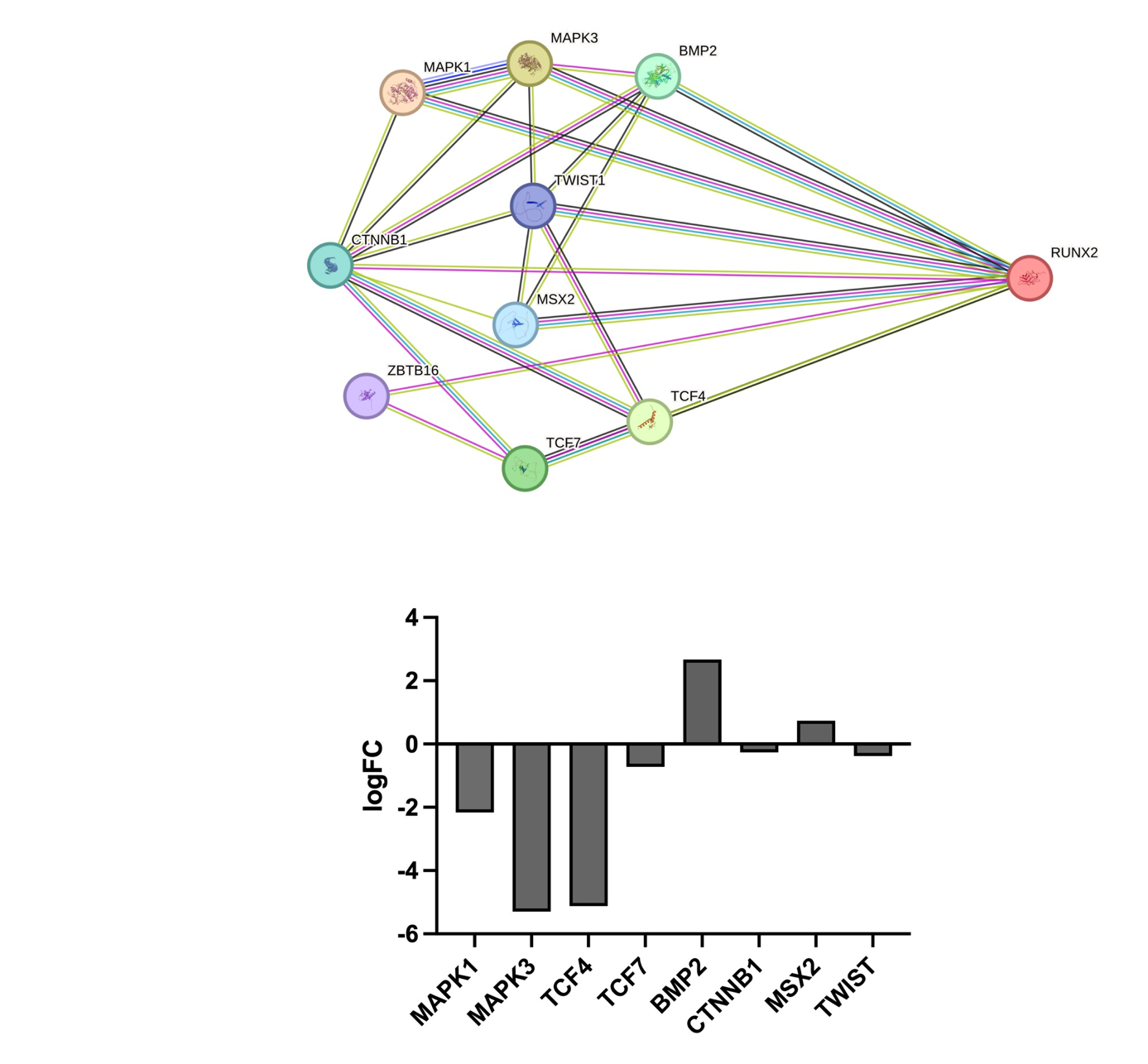


**FIGURE S1: Predicted upstream regulators of RUNX2 and their transcriptional response to FTI treatment**

The STRING network cluster shows the precomputed functional protein association network of the osteogenic genes and their gene neighbourhood. **(B)** Bar graph showing log2 fold changes (log2FC) in mRNA expression levels of selected RUNX2 upstream regulators in SaOS-2 cells treated with the farnesyltransferase inhibitor (FTI) Lonafarnib compared to control. Negative values indicate downregulation, positive values indicate upregulation.


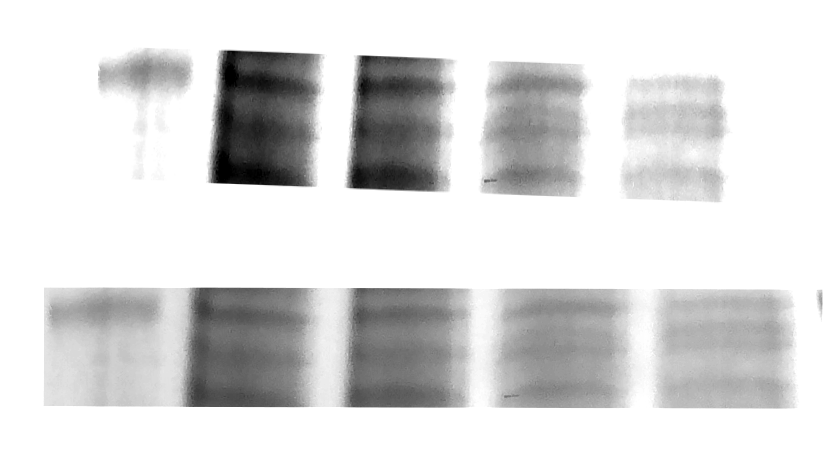


**70 kDa**

**Ladder M OM L (OM) T (OM)**

**FIGURE S2: Native SDS-PAGE after 96-hour treatment with 10 µM FTI**

SaOS-2 cells were treated with 10 µM of the indicated farnesyltransferase inhibitor for 96 hours. Cell lysates were analyzed by SDS-PAGE stained with coomassie blue. The figure shows representative results. M = Medium Group, OM = Osteogenic Medium, L (OM) = Lonafarnib treated in osteogenic medium, T (OM) = Tipifarnib treated in osteogenic medium.





**FIGURE S3:** **Heatmap of relative emPAI [%] in the molecular weight range of 66–72 kDa for the groups Control, Lonafarnib, and Tipifarnib.**

Shown are proteins detected in at least one of the treatment groups.. Grey boxes indicate undetected proteins. The color scale ranges from low (light grey) to high (green) relative abundance, normalized to the total proteome of each group.

C = Control, L = Lonafarnib, T = Tipifarnib

**Table S1: Proteins exclusively detected in control group**

| **Control** |
| --- |
| eukaryotic translation initiation factor 2 subunit 3 [Homo sapiens] |
| transmembrane protein 214 isoform 1 [Homo sapiens] |
| probable ATP-dependent RNA helicase DDX6 [Homo sapiens] |
| moesin isoform X2 [Homo sapiens] |
| eukaryotic translation initiation factor 3 subunit F [Homo sapiens] |
| rho GTPase-activating protein 44 isoform 1 [Homo sapiens] |
| nuclear receptor-binding protein isoform 1 [Homo sapiens] |
| 26S proteasome non-ATPase regulatory subunit 4 isoform 2 [Homo sapiens] |
| polypyrimidine tract-binding protein 1 isoform a [Homo sapiens] |
| mothers against decapentaplegic homolog 2 isoform 1 [Homo sapiens] |
| serine--tRNA ligase, cytoplasmic isoform a [Homo sapiens] |
| ribosomal protein S6 kinase beta-1 isoform a [Homo sapiens] |
| ABCF2-H2B protein [Homo sapiens] |
| asparagine--tRNA ligase, cytoplasmic [Homo sapiens] |
| T-complex protein 1 subunit zeta isoform a [Homo sapiens] |
| leucine zipper putative tumor suppressor 2 isoform a [Homo sapiens] |
| heterogeneous nuclear ribonucleoprotein D0 isoform c [Homo sapiens] |
| replication protein A 70 kDa DNA-binding subunit isoform 1 [Homo sapiens] |
| T-complex protein 1 subunit alpha isoform a [Homo sapiens] |
| acetoacetyl-CoA synthetase isoform 1 [Homo sapiens] |
| adseverin isoform 2 [Homo sapiens] |
| acetyl-coenzyme A synthetase, cytoplasmic isoform 1 [Homo sapiens] |
| DNA replication licensing factor MCM7 isoform 1 [Homo sapiens] |
| methylcrotonoyl-CoA carboxylase subunit alpha, mitochondrial isoform 1 precursor [Homo sapiens] |
| extended synaptotagmin-1 isoform 2 [Homo sapiens] |
| ran GTPase-activating protein 1 [Homo sapiens] |
| plastin-1 [Homo sapiens] |
| insulin-like growth factor 2 mRNA-binding protein 3 [Homo sapiens] |
| septin-7 isoform 1 [Homo sapiens] |
| tumor necrosis factor alpha-induced protein 2 isoform 1 [Homo sapiens] |
| myc box-dependent-interacting protein 1 isoform 8 [Homo sapiens] |
| heat shock protein HSP 90-alpha isoform 1 [Homo sapiens] |
| target of Myb protein 1 isoform 1 [Homo sapiens] |
| threonine--tRNA ligase 1, cytoplasmic isoform 1 [Homo sapiens] |
| histone deacetylase 2 [Homo sapiens] |
| protein arginine N-methyltransferase 5 isoform a [Homo sapiens] |
| ATP-dependent DNA helicase Q1 [Homo sapiens] |
| eukaryotic translation initiation factor 3 subunit D [Homo sapiens] |
| heterogeneous nuclear ribonucleoprotein K isoform a [Homo sapiens] |
| calcium-binding mitochondrial carrier protein Aralar1 [Homo sapiens] |
| adenylate kinase isoenzyme 5 isoform 2 [Homo sapiens] |
| SNW domain-containing protein 1 isoform X1 [Homo sapiens] |
| transmembrane protein 119 precursor [Homo sapiens] |
| acyl-coenzyme A thioesterase 8 [Homo sapiens] |
| kinesin light chain 1 isoform 1 [Homo sapiens] |
| RAS protein activator like-3 isoform 1 [Homo sapiens] |
| plasminogen activator inhibitor 1 RNA-binding protein isoform 4 [Homo sapiens] |
| abl interactor 1 isoform a [Homo sapiens] |
| tubulin alpha-1B chain [Homo sapiens] |
| lymphoid-restricted membrane protein isoform a [Homo sapiens] |
| U1 small nuclear ribonucleoprotein 70 kDa isoform 1 [Homo sapiens] |
| ubiquitin-associated protein 2-like isoform a [Homo sapiens] |
| spliceosome RNA helicase DDX39B [Homo sapiens] |
| ubiquilin-1 isoform 1 [Homo sapiens] |
| AP-2 complex subunit mu isoform a [Homo sapiens] |
| bifunctional 3'-phosphoadenosine 5'-phosphosulfate synthase 1 [Homo sapiens] |
| serine/threonine-protein kinase 4 isoform 1 [Homo sapiens] |
| eukaryotic initiation factor 4A-II [Homo sapiens] |
| protein LSM14 homolog A isoform b [Homo sapiens] |
| galectin-3-binding protein precursor [Homo sapiens] |
| calpain-1 catalytic subunit [Homo sapiens] |
| N-myc proto-oncogene protein isoform 1 [Homo sapiens] |
| F-box/LRR-repeat protein 18 isoform 2 [Homo sapiens] |
| TOM1-like protein 2 isoform 4 [Homo sapiens] |
| alpha-1,2-mannosyltransferase ALG9 isoform a [Homo sapiens] |
| calnexin isoform d precursor [Homo sapiens] |
| deoxynucleoside triphosphate triphosphohydrolase SAMHD1 isoform 1 [Homo sapiens] |
| nascent polypeptide-associated complex subunit alpha isoform b [Homo sapiens] |
| protocadherin-19 isoform b precursor [Homo sapiens] |
| peroxisomal membrane protein PEX14 [Homo sapiens] |
| DNA replication licensing factor MCM5 [Homo sapiens] |
| X-ray repair cross-complementing protein 5 [Homo sapiens] |
| heterogeneous nuclear ribonucleoprotein R isoform 1 [Homo sapiens] |
| HEAT repeat-containing protein 3 isoform a [Homo sapiens] |
| ubiquitin-40S ribosomal protein S27a precursor [Homo sapiens] |
| Y-box-binding protein 1 [Homo sapiens] |
| methionine aminopeptidase 2 isoform 1 [Homo sapiens] |
| eukaryotic translation initiation factor 3 subunit G [Homo sapiens] |
| iporin isoform 1 [Homo sapiens] |
| eukaryotic translation initiation factor 2A isoform 1 [Homo sapiens] |
| transitional endoplasmic reticulum ATPase isoform 1 [Homo sapiens] |
| ras GTPase-activating-like protein IQGAP2 isoform 1 [Homo sapiens] |
| far upstream element-binding protein 1 isoform 2 [Homo sapiens] |
| ATP-dependent 6-phosphofructokinase, muscle type isoform 2 [Homo sapiens] |
| glycylpeptide N-tetradecanoyltransferase 1 [Homo sapiens] |
| xaa-Pro dipeptidase isoform 1 [Homo sapiens] |
| abl interactor 2 isoform c [Homo sapiens] |
| CTP synthase 1 isoform a [Homo sapiens] |
| interferon-induced, double-stranded RNA-activated protein kinase isoform a [Homo sapiens] |
| transcription factor Sp7 isoform a [Homo sapiens] |
| pre-mRNA-splicing factor ATP-dependent RNA helicase DHX15 [Homo sapiens] |
| elongation factor 2 [Homo sapiens] |
| tubulin beta chain isoform a [Homo sapiens] |
| fragile X mental retardation syndrome-related protein 1 isoform a [Homo sapiens] |
| long-chain-fatty-acid--CoA ligase 4 isoform 1 [Homo sapiens] |
| HLA class I histocompatibility antigen, A alpha chain A precursor [Homo sapiens] |
| heterogeneous nuclear ribonucleoprotein L isoform a [Homo sapiens] |
| adenylyl cyclase-associated protein 1 isoform a [Homo sapiens] |
| THUMP domain-containing protein 3 [Homo sapiens] |
| threonine--tRNA ligase, mitochondrial isoform a [Homo sapiens] |
| gelsolin isoform b [Homo sapiens] |
| WD repeat-containing protein 1 isoform 2 [Homo sapiens] |
| ubiquitin-like modifier-activating enzyme 1 [Homo sapiens] |

**Table S2: Proteins exclusively detected in Lonafarnib group**

| **Lonafarnib** |
| --- |
| 60S ribosomal protein L29 [Homo sapiens] |
| GATOR complex protein WDR59 isoform X3 [Homo sapiens] |
| keratin, type II cuticular Hb1 [Homo sapiens] |
| serine/threonine-protein phosphatase 2A 56 kDa regulatory subunit delta isoform isoform 1 [Homo sapiens] |
| 40S ribosomal protein S3 isoform 1 [Homo sapiens] |
| 60S ribosomal protein L19 isoform 1 [Homo sapiens] |
| protein phosphatase 1 regulatory subunit 3A [Homo sapiens] |
| 28S ribosomal protein S31, mitochondrial [Homo sapiens] |
| vacuole membrane protein 1 isoform 1 [Homo sapiens] |
| keratin, type II cytoskeletal 6B [Homo sapiens] |
| mannosyl-oligosaccharide 1,2-alpha-mannosidase IA [Homo sapiens] |
| bromodomain and WD repeat-containing protein 1 isoform X5 [Homo sapiens] |
| keratin, type I cytoskeletal 14 [Homo sapiens] |
| 60S ribosomal protein L7a [Homo sapiens] |
| DNA-directed RNA polymerases I and III subunit RPAC1 isoform 1 [Homo sapiens] |
| surfeit locus protein 4 isoform 1 [Homo sapiens] |
| ATPase family AAA domain-containing protein 3A isoform 1 [Homo sapiens] |
| keratin, type I cytoskeletal 16 [Homo sapiens] |
| transmembrane protein 109 precursor [Homo sapiens] |
| stromal interaction molecule 2 isoform 2 precursor [Homo sapiens] |
| tropomodulin-1 [Homo sapiens] |
| protein strawberry notch homolog 2 isoform 1 [Homo sapiens] |
| DEP domain-containing protein 1A isoform a [Homo sapiens] |
| golgin subfamily B member 1 isoform 2 [Homo sapiens] |
| superoxide dismutase [Mn], mitochondrial isoform A precursor [Homo sapiens] |
| glutamate receptor ionotropic, kainate 5 isoform X7 [Homo sapiens] |
| protocadherin Fat 1 precursor [Homo sapiens] |
| V-type proton ATPase subunit S1 precursor [Homo sapiens] |

**Table S3: Proteins exclusively detected in Tipifarnib group**

| **Tipifarnib** |
| --- |
| myosin-4 [Homo sapiens] |
| ras-related protein Rab-34 isoform X1 [Homo sapiens] |
| cyclin-J isoform X5 [Homo sapiens] |
| homeobox protein Hox-D9 [Homo sapiens] |
| vacuolar protein sorting-associated protein 26A isoform 1 [Homo sapiens] |
| synaptotagmin-7 isoform 3 [Homo sapiens] |
| proprotein convertase subtilisin/kexin type 4 isoform X12 [Homo sapiens] |
| mitochondrial inner membrane protein OXA1L [Homo sapiens] |
| thioredoxin domain-containing protein 5 isoform 1 precursor [Homo sapiens] |
| sorbin and SH3 domain-containing protein 1 isoform 6 [Homo sapiens] |
| ATPase family AAA domain-containing protein 3A isoform 2 [Homo sapiens] |
| protein KRI1 homolog [Homo sapiens] |
| tetraspanin-3 isoform 1 [Homo sapiens] |
| protein FAM110D [Homo sapiens] |
| dynein regulatory complex subunit 2 isoform 1 [Homo sapiens] |
| dystrophin isoform Dp427c [Homo sapiens] |
| CD99 antigen isoform a precursor [Homo sapiens] |
| lactadherin isoform a preproprotein [Homo sapiens] |
| 40S ribosomal protein SA isoform 1 [Homo sapiens] |
| coagulation factor V preproprotein [Homo sapiens] |
| limbic system-associated membrane protein isoform 1 preproprotein [Homo sapiens] |
| DISP complex protein LRCH3 isoform X4 [Homo sapiens] |
| ubiquilin-like protein [Homo sapiens] |
| NGFI-A-binding protein 1 isoform 1 [Homo sapiens] |
| centrosome-associated protein 350 [Homo sapiens] |
| translocon-associated protein subunit alpha isoform 1 precursor [Homo sapiens] |
| butyrophilin subfamily 2 member A1 isoform 1 precursor [Homo sapiens] |
| ATP synthase subunit beta, mitochondrial precursor [Homo sapiens] |
| heterogeneous nuclear ribonucleoprotein Q isoform 2 [Homo sapiens] |
| beta-2-glycoprotein 1 precursor [Homo sapiens] |
| prosaposin isoform a preproprotein [Homo sapiens] |

**Table S4: Proteins exclusively detected in control & Lonafarnib groups**

| **Control & Lonafarnib** |
| --- |
| cleavage and polyadenylation specificity factor subunit 7 isoform 1 [Homo sapiens] |
| T-complex protein 1 subunit eta isoform a [Homo sapiens] |
| pyruvate kinase PKM isoform a [Homo sapiens] |
| non-POU domain-containing octamer-binding protein isoform 1 [Homo sapiens] |
| mini-chromosome maintenance complex-binding protein isoform 1 [Homo sapiens] |
| proteasomal ubiquitin receptor ADRM1 isoform 1 [Homo sapiens] |
| cytospin-B isoform 3 [Homo sapiens] |
| 60S ribosomal protein L5 [Homo sapiens] |
| heterogeneous nuclear ribonucleoprotein Q isoform 3 [Homo sapiens] |
| dynamin-1-like protein isoform 3 [Homo sapiens] |
| septin-9 isoform c [Homo sapiens] |
| tyrosine-protein phosphatase non-receptor type 11 isoform 1 [Homo sapiens] |
| E3 ubiquitin/ISG15 ligase TRIM25 [Homo sapiens] |
| heat shock protein HSP 90-beta isoform a [Homo sapiens] |
| endoplasmin precursor [Homo sapiens] |
| autophagy-related protein 16-1 isoform 2 [Homo sapiens] |
| negative elongation factor B [Homo sapiens] |
| glucosidase 2 subunit beta isoform 1 precursor [Homo sapiens] |
| bifunctional UDP-N-acetylglucosamine 2-epimerase/N-acetylmannosamine kinase isoform 2 [Homo sapiens] |
| serine/threonine-protein kinase Nek5 isoform X4 [Homo sapiens] |
| nuclear autoantigenic sperm protein isoform 2 [Homo sapiens] |
| hepatoma-derived growth factor isoform a [Homo sapiens] |

**Table S5: Proteins exclusively detected in control & Tipifarnib groups**

| **Control & Tipifarnib** |
| --- |
| protein piccolo isoform 2 [Homo sapiens] |
| ankyrin repeat and SAM domain-containing protein 1A [Homo sapiens] |
| testis-specific Y-encoded protein 1 isoform TSPY-S [Homo sapiens] |
| fibroblast growth factor-binding protein 2 precursor [Homo sapiens] |
| phosphoglycerate kinase 1 [Homo sapiens] |
| mucin-19 precursor [Homo sapiens] |
| annexin A6 isoform 1 [Homo sapiens] |
| integrin alpha-4 isoform 1 preproprotein [Homo sapiens] |
| chymotrypsin-like elastase family member 3A preproprotein [Homo sapiens] |
| lanosterol synthase isoform 1 [Homo sapiens] |
| immunoglobulin superfamily member 8 precursor [Homo sapiens] |
| prolyl endopeptidase [Homo sapiens] |
| vesicle-fusing ATPase [Homo sapiens] |
| spectrin beta chain, non-erythrocytic 2 [Homo sapiens] |
| prolyl 4-hydroxylase subunit alpha-1 isoform 1 precursor [Homo sapiens] |
| apoptosis-inducing factor 1, mitochondrial isoform AIF precursor [Homo sapiens] |
| phosphoglucomutase-2 [Homo sapiens] |

**Table S6: Proteins exclusively detected in Lonafarnib & Tipifarnib groups**

| **Lonafarnib & Tipifarnib** |
| --- |
| mitofusin-2 [Homo sapiens] |
| secretory carrier-associated membrane protein 3 isoform 1 [Homo sapiens] |
| ephrin type-A receptor 2 isoform 1 precursor [Homo sapiens] |
| insulin-like growth factor 2 mRNA-binding protein 1 isoform 1 [Homo sapiens] |
| calcium-binding mitochondrial carrier protein Aralar2 isoform 2 [Homo sapiens] |
| N-acetylgalactosaminyltransferase 7 isoform 1 [Homo sapiens] |
| sodium/potassium-transporting ATPase subunit beta-1 [Homo sapiens] |
| inaD-like protein isoform 1 [Homo sapiens] |
| histone H1.5 [Homo sapiens] |
| beta-glucuronidase isoform 1 precursor [Homo sapiens] |
| dihydrolipoyllysine-residue succinyltransferase component of 2-oxoglutarate dehydrogenase complex, mitochondrial isoform 1 precursor [Homo sapiens] |
| keratin, type II cytoskeletal 5 [Homo sapiens] |
| HLA class I histocompatibility antigen, C alpha chain precursor [Homo sapiens] |
| keratin, type II cytoskeletal 2 epidermal [Homo sapiens] |
| NADPH--cytochrome P450 reductase [Homo sapiens] |
| large neutral amino acids transporter small subunit 1 [Homo sapiens] |
| tubulin alpha-4A chain isoform 1 [Homo sapiens] |
| tubulin beta chain isoform b [Homo sapiens] |
| GPI transamidase component PIG-T isoform 1 precursor [Homo sapiens] |
| nesprin-1 isoform 2 [Homo sapiens] |
| cathepsin D preproprotein [Homo sapiens] |
| aspartate aminotransferase, mitochondrial isoform 1 precursor [Homo sapiens] |
| midasin [Homo sapiens] |
| phosphoenolpyruvate carboxykinase [GTP], mitochondrial isoform 1 precursor [Homo sapiens] |
| EGF-like repeat and discoidin I-like domain-containing protein 3 isoform 1 precursor [Homo sapiens] |
| gelsolin isoform a precursor [Homo sapiens] |
| catenin beta-1 isoform 1 [Homo sapiens] |
| lipase member K isoform X8 [Homo sapiens] |
| ceramide synthase 2 [Homo sapiens] |
| beta-actin-like protein 2 [Homo sapiens] |
| adenosylhomocysteinase isoform 1 [Homo sapiens] |
| solute carrier family 35 member F6 precursor [Homo sapiens] |

**Table S7: Proteins exclusively detected in all three groups**

| **Control, Lonafarnib & Tipifarnib** |
| --- |
| alkaline phosphatase, tissue-nonspecific isozyme isoform 1 preproprotein [Homo sapiens] |
| V-type proton ATPase catalytic subunit A [Homo sapiens] |
| phospholipase D3 [Homo sapiens] |
| heat shock 70 kDa protein 1A [Homo sapiens] |
| transketolase isoform 1 [Homo sapiens] |
| peptidyl-prolyl cis-trans isomerase FKBP9 isoform 1 precursor [Homo sapiens] |
| glycerol-3-phosphate dehydrogenase, mitochondrial precursor [Homo sapiens] |
| glutaminase kidney isoform, mitochondrial isoform 1 precursor [Homo sapiens] |
| protein transport protein Sec23A [Homo sapiens] |
| lamin-B2 [Homo sapiens] |
| protein kinase C and casein kinase substrate in neurons protein 2 isoform A [Homo sapiens] |
| histone H1.2 [Homo sapiens] |
| KH domain-containing, RNA-binding, signal transduction-associated protein 1 isoform 1 [Homo sapiens] |
| reticulon-4 isoform A [Homo sapiens] |
| protein disulfide-isomerase A6 isoform d precursor [Homo sapiens] |
| heterogeneous nuclear ribonucleoprotein M isoform a [Homo sapiens] |
| protein disulfide-isomerase A4 isofrom 2 precursor [Homo sapiens] |
| protein O-glucosyltransferase 3 isoform 1 precursor [Homo sapiens] |
| protein ERGIC-53 precursor [Homo sapiens] |
| protein PRRC1 isoform 2 [Homo sapiens] |
| lamin isoform C [Homo sapiens] |
| sphingomyelin phosphodiesterase 3 [Homo sapiens] |
| lysosomal acid glucosylceramidase isoform 1 precursor [Homo sapiens] |
| 60S ribosomal protein L3 isoform a [Homo sapiens] |
| protein disulfide-isomerase precursor [Homo sapiens] |
| probable ATP-dependent RNA helicase DDX5 isoform a [Homo sapiens] |
| transferrin receptor protein 1 isoform 1 [Homo sapiens] |
| delta-1-pyrroline-5-carboxylate synthase isoform 1 [Homo sapiens] |
| DDRGK domain-containing protein 1 [Homo sapiens] |
| cytoskeleton-associated protein 4 [Homo sapiens] |
| nucleobindin-2 isoform 1 preproprotein [Homo sapiens] |
| stress-70 protein, mitochondrial precursor [Homo sapiens] |
| HERV-H LTR-associating protein 3 isoform X1 [Homo sapiens] |
| long-chain-fatty-acid--CoA ligase 3 [Homo sapiens] |
| ATP-dependent Clp protease ATP-binding subunit clpX-like, mitochondrial precursor [Homo sapiens] |
| vimentin [Homo sapiens] |
| stress-induced-phosphoprotein 1 isoform b [Homo sapiens] |
| lysosomal acid phosphatase isoform 1 precursor [Homo sapiens] |
| minor histocompatibility antigen H13 isoform 1 [Homo sapiens] |
| calreticulin precursor [Homo sapiens] |
| dedicator of cytokinesis protein 8 isoform 1 [Homo sapiens] |
| keratin, type I cytoskeletal 9 [Homo sapiens] |
| leucine-rich repeat-containing protein 47 [Homo sapiens] |
| cytoplasmic dynein 1 light intermediate chain 1 isoform 1 [Homo sapiens] |
| glutamate dehydrogenase 1, mitochondrial isoform a precursor [Homo sapiens] |
| annexin A11 isoform 1 [Homo sapiens] |
| plastin-3 isoform 1 [Homo sapiens] |
| 60S ribosomal protein L6 isoform 1 [Homo sapiens] |
| dolichyl-diphosphooligosaccharide--protein glycosyltransferase subunit 1 precursor [Homo sapiens] |
| X-ray repair cross-complementing protein 6 isoform 1 [Homo sapiens] |
| Golgi reassembly-stacking protein 2 isoform 1 [Homo sapiens] |
| peptidyl-prolyl cis-trans isomerase FKBP10 precursor [Homo sapiens] |
| actin, cytoplasmic 1 [Homo sapiens] |
| elongation factor 1-gamma [Homo sapiens] |
| lysine--tRNA ligase isoform 2 [Homo sapiens] |
| procollagen galactosyltransferase 1 precursor [Homo sapiens] |
| trifunctional enzyme subunit beta, mitochondrial isoform 1 precursor [Homo sapiens] |
| calumenin isoform a precursor [Homo sapiens] |
| serine/threonine-protein kinase PAK 2 [Homo sapiens] |
| CCR4-NOT transcription complex subunit 2 [Homo sapiens] |
| multifunctional procollagen lysine hydroxylase and glycosyltransferase LH3 precursor [Homo sapiens] |
| cation-dependent mannose-6-phosphate receptor isoform 1 precursor [Homo sapiens] |
| dolichyl-diphosphooligosaccharide--protein glycosyltransferase 48 kDa subunit precursor [Homo sapiens] |
| peroxisomal multifunctional enzyme type 2 isoform 2 [Homo sapiens] |
| 60S ribosomal protein L7 isoform 1 [Homo sapiens] |
| glyceraldehyde-3-phosphate dehydrogenase isoform 1 [Homo sapiens] |
| glutamine--fructose-6-phosphate aminotransferase [isomerizing] 1 isoform 2 [Homo sapiens] |
| elongation factor G, mitochondrial isoform 2 [Homo sapiens] |
| ATP synthase subunit alpha, mitochondrial isoform a precursor [Homo sapiens] |
| heat shock cognate 71 kDa protein isoform 1 [Homo sapiens] |
| signal recognition particle subunit SRP68 isoform 1 [Homo sapiens] |
| alkyldihydroxyacetonephosphate synthase, peroxisomal precursor [Homo sapiens] |
| nucleobindin-1 precursor [Homo sapiens] |
| xaa-Pro aminopeptidase 1 isoform 1 [Homo sapiens] |
| keratin, type I cytoskeletal 10 isoform 1 [Homo sapiens] |
| lamin-B1 isoform 1 [Homo sapiens] |
| succinate dehydrogenase [ubiquinone] flavoprotein subunit, mitochondrial isoform 1 [Homo sapiens] |
| sodium/potassium-transporting ATPase subunit beta-3 [Homo sapiens] |
| endoplasmic reticulum chaperone BiP precursor [Homo sapiens] |
| tubulin beta-4B chain [Homo sapiens] |
| elongation factor 1-alpha 1 [Homo sapiens] |
| electron transfer flavoprotein-ubiquinone oxidoreductase, mitochondrial isoform 1 precursor [Homo sapiens] |
| alpha-2-HS-glycoprotein isoform 2 preproprotein [Homo sapiens] |
| carnitine O-palmitoyltransferase 2, mitochondrial isoform 1 precursor [Homo sapiens] |
| trifunctional enzyme subunit alpha, mitochondrial precursor [Homo sapiens] |
| GMP synthase [glutamine-hydrolyzing] [Homo sapiens] |
| phenylalanine--tRNA ligase beta subunit [Homo sapiens] |
| acylamino-acid-releasing enzyme [Homo sapiens] |
| procollagen-lysine,2-oxoglutarate 5-dioxygenase 1 isoform 2 precursor [Homo sapiens] |
| lactotransferrin isoform 1 preproprotein [Homo sapiens] |
| neurolysin, mitochondrial [Homo sapiens] |
| long-chain-fatty-acid--CoA ligase 1 isoform a [Homo sapiens] |
| adipocyte plasma membrane-associated protein [Homo sapiens] |
| basigin isoform 1 precursor [Homo sapiens] |
| polyadenylate-binding protein 1 [Homo sapiens] |
| arginine--tRNA ligase, cytoplasmic [Homo sapiens] |
| nuclear protein localization protein 4 homolog isoform 1 [Homo sapiens] |
| CDK5 regulatory subunit-associated protein 3 isoform b [Homo sapiens] |
| sec1 family domain-containing protein 1 isoform a [Homo sapiens] |
| dihydropyrimidinase-related protein 2 isoform 2 [Homo sapiens] |
| ATP-dependent 6-phosphofructokinase, liver type isoform b [Homo sapiens] |
| NHL repeat-containing protein 2 [Homo sapiens] |
| heat shock protein 75 kDa, mitochondrial isoform 1 precursor [Homo sapiens] |
| tropomyosin alpha-1 chain isoform Tpm1.5cy [Homo sapiens] |
| serine hydroxymethyltransferase, mitochondrial isoform 1 precursor [Homo sapiens] |
| 60 kDa heat shock protein, mitochondrial [Homo sapiens] |
| protein disulfide-isomerase A3 precursor [Homo sapiens] |
| T-complex protein 1 subunit gamma isoform a [Homo sapiens] |
| formin-like protein 1 isoform X1 [Homo sapiens] |
| N-acetyltransferase 9 isoform 12 [Homo sapiens] |
| sequestosome-1 isoform 1 [Homo sapiens] |
| propionyl-CoA carboxylase alpha chain, mitochondrial isoform a precursor [Homo sapiens] |
| ERO1-like protein alpha precursor [Homo sapiens] |
| serum albumin preproprotein [Homo sapiens] |
| alpha-enolase isoform 1 [Homo sapiens] |
| keratin, type II cytoskeletal 1 [Homo sapiens] |
| matrix-remodeling-associated protein 7 isoform 3 precursor [Homo sapiens] |
| bifunctional purine biosynthesis protein PURH [Homo sapiens] |
